# Chemosynthetic and photosynthetic bacteria contribute differentially to primary production across a steep desert aridity gradient

**DOI:** 10.1038/s41396-021-01001-0

**Published:** 2021-05-25

**Authors:** Sean K. Bay, David W. Waite, Xiyang Dong, Osnat Gillor, Steven L. Chown, Philip Hugenholtz, Chris Greening

**Affiliations:** 1 Department of Microbiology, Biomedicine Discovery Institute, Monash University, Clayton, VIC Australia; 2 School of Biological Sciences, Monash University, Clayton, VIC Australia; 3 Australian Centre for Ecogenomics, School of Chemistry and Molecular Biosciences, University of Queensland, St Lucia, QLD Australia; 4 School of Biological Sciences, University of Auckland, Auckland, New Zealand; 5 School of Marine Sciences, Sun Yat-Sen University, Zhuhai, China; 6 Zuckerberg Institute for Water Research, Blaustein Institutes for Desert Research, Ben Gurion University of the Negev, Sde Boker, Israel

**Keywords:** Microbial ecology, Biogeochemistry

## Abstract

Desert soils harbour diverse communities of aerobic bacteria despite lacking substantial organic carbon inputs from vegetation. A major question is therefore how these communities maintain their biodiversity and biomass in these resource-limiting ecosystems. Here, we investigated desert topsoils and biological soil crusts collected along an aridity gradient traversing four climatic regions (sub-humid, semi-arid, arid, and hyper-arid). Metagenomic analysis indicated these communities vary in their capacity to use sunlight, organic compounds, and inorganic compounds as energy sources. Thermoleophilia, Actinobacteria, and Acidimicrobiia were the most abundant and prevalent bacterial classes across the aridity gradient in both topsoils and biocrusts. Contrary to the classical view that these taxa are obligate organoheterotrophs, genome-resolved analysis suggested they are metabolically flexible, with the capacity to also use atmospheric H_2_ to support aerobic respiration and often carbon fixation. In contrast, Cyanobacteria were patchily distributed and only abundant in certain biocrusts. Activity measurements profiled how aerobic H_2_ oxidation, chemosynthetic CO_2_ fixation, and photosynthesis varied with aridity. Cell-specific rates of atmospheric H_2_ consumption increased 143-fold along the aridity gradient, correlating with increased abundance of high-affinity hydrogenases. Photosynthetic and chemosynthetic primary production co-occurred throughout the gradient, with photosynthesis dominant in biocrusts and chemosynthesis dominant in arid and hyper-arid soils. Altogether, these findings suggest that the major bacterial lineages inhabiting hot deserts use different strategies for energy and carbon acquisition depending on resource availability. Moreover, they highlight the previously overlooked roles of Actinobacteriota as abundant primary producers and trace gases as critical energy sources supporting productivity and resilience of desert ecosystems.

## Introduction

Photosynthetic primary producers are in low abundance in the desert and dryland ecosystems that span 40% of the earth’s surface [1]. Whereas most terrestrial ecosystems are driven by plant-derived organic matter, plant growth declines with aridity, and vegetation is particularly sparse in arid and hyper-arid deserts [2, 3]. Some cyanobacteria and microalgae can nevertheless persist even in hyper-arid deserts by retreating to environmental refugia such as biological soil crusts (biocrusts) and lithic niches [4–11]; such environments provide desiccation buffers and protection from ultraviolet radiation [9, 12–15], with the surface stability and physical structure of biocrusts also favouring moisture retention [16–18]. Occasional precipitation events and transient moisture, for example from early morning dewfall, are proposed to serve as sufficient inputs to activate photosynthetic activity [18–20]. As oxygenic photoautotrophs, these microorganisms use photosystems to capture light and transduce energy, and use either type IA or IB RuBisCO (ribulose 1,5-bisphosphate carboxylase / oxygenase) within the Calvin-Benson-Bassham cycle to fix carbon dioxide (CO_2_) into organic carbon [21, 22]. In the interior of arid and hyper-arid deserts, photoautotrophic communities become increasingly rare and spatially fragmented [4, 9, 23, 24]. Diverse communities of microorganisms can nevertheless be found in bare desert soils, where they survive the cumulative pressures of low water and carbon availability, elevated temperatures, salinity, and ultraviolet radiation [25–30]. The most abundant microorganisms in these environments are members of dominant bacterial soil phyla such as Actinobacteriota, Proteobacteria, and Chloroflexota, and are typically thought to be aerobic obligate organoheterotrophs [20, 25, 28, 31–33]. A major question is how these bacteria maintain their energy and carbon needs in these environments given their multiple physicochemical pressures and the dearth of photosynthetic primary producers.

Within desert environments, bacteria transition between growing and dormant states depending on the availability of water and other resources [20, 32, 34, 35]. Dormancy is a life history strategy in which cells enter a reversible state of reduced metabolic activity and increased environmental resilience in response to pressures such as resource limitation [36, 37]. In turn, by allowing bacteria to persist under conditions which favour survival over growth, dormancy is thought to enhance microbial richness and ecosystem resilience [38–40]. While the energy needs of dormant cells are usually three orders of magnitude lower than growing cells, some energy is nevertheless necessary for cells to maintain basic functions, allowing an eventual return to an active state [36, 37]. It is generally thought that desert bacteria primarily survive in a dormant state by using macromolecular reserves, which are synthesized when organic carbon becomes transiently available following hydration events [35, 41]. Recent culture-based studies have demonstrated, however, that some aerobic organoheterotrophs in fact broaden their repertoire of exogenous substrates during carbon starvation. Most notably, various bacterial isolates can use the atmospheric trace gases hydrogen (H_2_) and carbon monoxide (CO) as alternative electron donors to sustain aerobic respiration [42–50]. Genetic studies focusing on Actinobacteriota have shown that trace gas oxidation significantly increases long-term survival under energy starvation [51–54]. Although these studies did not focus on desert isolates, it is plausible that Actinobacteriota and other taxa in desert ecosystems also meet their energy needs by scavenging trace gases in their dormant states.

Recent metagenomic and biogeochemical studies have implicated atmospheric H_2_ as a particularly important energy source driving aerobic respiration and carbon fixation in desert environments [11, 32, 55]. This gas is thought to be highly dependable for bacteria for four key reasons: (i) it is ubiquitous throughout the earth’s lower atmosphere (mixing ratio 0.53 ppmv), (ii) it readily diffuses through cell membranes, (iii) it has a low activation energy, and (iv) its combustion yields a high amount of free energy [56–58]. Atmospheric H_2_ oxidation yields ~2.0 × 10^−15^ W per cell across diverse ecosystems [59], which meets maintenance energy requirements for most bacterial pure cultures [60–63] and exceeds those of highly oligotrophic ecosystems (10^−17^ to 10^−19^ W per cell) [64–66]. It has also been proposed that atmospheric H_2_ oxidation enables bacteria to allocate more organic carbon for anabolism rather than catabolism [67]. Bacteria oxidize atmospheric H_2_ using high-affinity, oxygen-tolerant [NiFe]-hydrogenases; these bacteria transfer electrons derived from H_2_ through the quinone pool to terminal oxidases, resulting in the generation of proton-motive force [43, 68, 69]. Various hydrogenase lineages are known to support aerobic H_2_ oxidation, including the group 1h, 1d, 1f, 1l, and 2a [NiFe]-hydrogenases [43, 49, 70–73], the first of which seems to be principally responsible for atmospheric H_2_ oxidation in soil ecosystems [42, 43, 47, 59, 74]. Some aerobic bacteria can use electrons derived from H_2_ to fix CO_2_ into biomass [75–77]. Aerobic hydrogenotrophic growth was conventionally thought to be restricted to H_2_-enriched environments such as nitrogen-fixing root nodules of legumes and geothermal systems [75]. Recent studies have suggested, however, that desert soil bacteria use atmospheric H_2_ as an energy source to support carbon fixation. Genome-resolved metagenomic analysis has revealed that multiple bacterial phyla, including Actinobacteriota, co-encode group 1h and 1l [NiFe]-hydrogenases together with a type IE RuBisCO linked to the Calvin-Benson-Bassam cycle [11, 55, 73, 78]. Consistently, these desert soils rapidly oxidised atmospheric H_2_ and fixed CO_2_ into biomass, through a process described as atmospheric chemosynthesis [55, 73]. Hot desert soils also mediate atmospheric H_2_ trace gas oxidation, in a process significantly stimulated together with photosynthesis following precipitation pulses, though it remains unclear whether such soils are also capable of hydrogenotrophic carbon fixation [32, 59]. Altogether, this suggests that atmospheric trace gases provide a dependable means for energy generation and potentially a minimalistic strategy for primary production in extreme desert environments [20, 79].

In this study, we build on these advances to better resolve the relative contributions of photosynthetic and chemosynthetic processes to desert primary production. We sampled topsoils and biocrusts along a steep aridity gradient in the Negev Desert (Israel) traversing sub-humid, semi-arid, arid, and hyper-arid climatic zones. We predict that oxygenic photosynthesis should predominate in biocrusts and wetter soils, whereas trace gas oxidation should predominate in drier soils. Here we performed side-by-side metagenomic and biogeochemical profiling to test this prediction.

## Materials and methods

### Field sampling

**Fig. 1:**
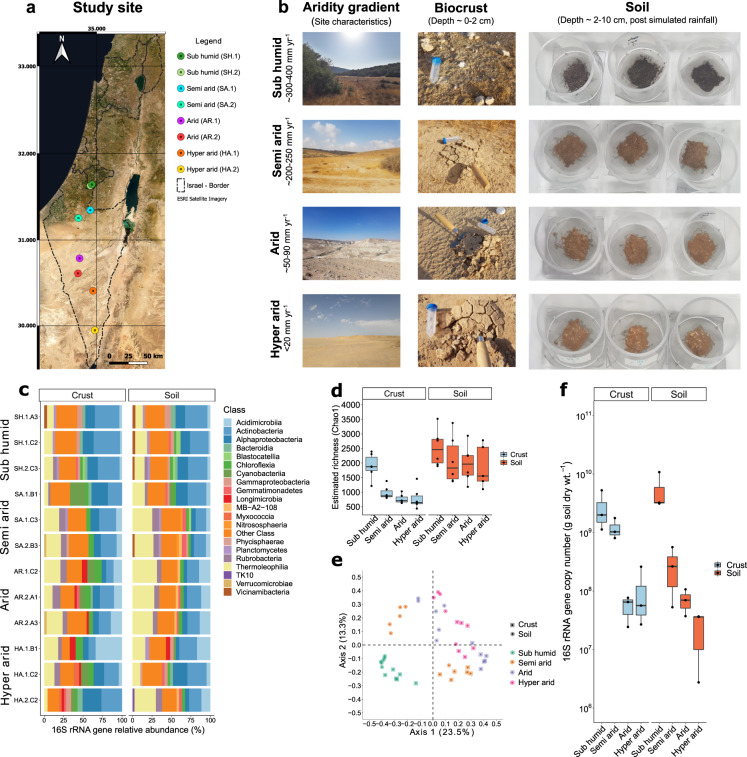
Microbial community composition, diversity, and abundance of biocrust and topsoil samples collected across the Israel aridity gradient. Results are shown for biocrust and topsoil samples collected for each climatic zone. **a** Study site showing satellite imagery basemap with country administrative borders (black dashes) and site geolocations. **b** Images depict (from left to right) typical site characteristics, biocrust appearance during sample collection, and soil sample appearance following simulated rainfall. **c** Stacked barchart showing class-level bacterial and archaeal composition based on metagenomic reads of the 16S rRNA gene. Taxonomic classification follows GTDB taxonomy. **d** Boxplot showing the richness estimate (Chao1) of soil and biocrust communities along the aridity gradient based on 16S rRNA gene amplicon sequencing. **e** Beta diversity (Bray-Curtis) PCoA ordination visualizing differences in soil and crust community composition between the four climatic zones based on 16S rRNA gene amplicon sequencing. **f** Boxplot showing 16S rRNA gene copy number for both crust and topsoil along the aridity gradient. Community composition based on 16S rRNA gene amplicon sequencing and richness/beta diversity based on metagenomic sequencing are shown for comparison in Fig. S2.

The sampling transect extended for 160 km in a north/south direction across the Judea Hills and Negev Desert regions of Israel (Fig. 1a). Samples were collected from four climatic zones differentiated by mean annual precipitation patterns: sub-humid shrubland (300–400 mm yr^−1^), semi-arid grassland (~200–250 mm yr^−1^), arid desert (~50–90 mm yr^−1^), and hyper-arid desert (<20 mm yr^−1^) [30]. Biocrust and soil samples were collected according to a previously described hierarchical sampling design (4 zones × 2 sites × 3 plots × 3 subsamples) [30]. Sampling was conducted over a 10-day period in May 2017. To minimize the effects of non-climatic variables, sampling was restricted to wind-deposited loessic soils in the sub-humid, semi-arid, and arid zone, and gypsum soils in the hyper-arid zone. In addition, all samples collected were vegetation-free, contained visible biocrusts, and were at least 100 m from roads and slopes. Biocrust samples (0–2 cm depth) were extracted whole using a stainless steel spatula to separate the biocrust layer from the underling soil (Fig. 1b). Reflecting high organic matter and carbon content, sub-humid sites harboured dark brown crusts, which were replaced by lighter colours with increasing aridity; some semi-arid and arid crusts had dark brown speckles, indicating abundant cyanobacterial communities. Biocrust samples were transferred to fill a petri dish (60 mm diameter) padded with cotton wool and sealed using parafilm. Underlying topsoils (2–10 cm) were also sampled and transferred to screw top Falcon tube (50 ml). Within 24 h of sampling, all soils were homogenised by sieving (500 µm) and soil water content (as a percentage) was measured gravimetrically in duplicate. All samples were stored at 4 °C and subject to physicochemical analysis as previously described [30]. For this study, the three subsamples per plot were pooled to yield 48 biologically independent samples, specifically six topsoils and six biocrust samples from each of the four climatic zones (Table S1).

### 16S rRNA gene amplicon sequencing

Total community DNA was extracted from each of the 48 independent samples and subject to 16S rRNA gene amplicon sequencing. DNA was extracted from 0.25 g of sample using the MoBio PowerSoil Isolation Kit according to the manufacturer’s instructions. Samples were eluted in DNase- and RNase-free UltraPure Water (ThermoFisher). A sample-free negative control was also run. Nucleic acid purity and yield were confirmed using a Nanodrop 1000 and Qubit Fluorometer respectively. All 48 independent samples were subject to 16S rRNA gene sequencing. For each sample, the V4 hypervariable region of the 16S rRNA gene was amplified using the universal Earth Microbiome Project primers F515 and R806. MiSeq (Illumina) pair-end sequencing was performed at the Australian Centre for Ecogenomics, University of Queensland. Raw reads were demultiplexed and subject to primer removal, quality filtering, and denoising using the DADA2 pipeline [80]. Representative reads were mapped to the SILVA database (release 138) [81]. After denoising, a total of 22,519 amplicon sequence variants (ASVs) were retained for the community and diversity analysis. An average of 81,793 sequences and 1585 ASVs were detected per sample, whereas the extraction blank yielded just four sequences and two ASVs.

### Quantitative PCR

Quantitative polymerase chain reactions (qPCR) were used to estimate total bacterial and archaeal biomass of biocrust and topsoil samples. The 16S rRNA gene was amplified using the degenerate primer pair (515 F 5ʹ-154 GTGYCAGCMGCCGCGGTAA-3ʹ and 806 R 5ʹ-GGACTACNVGGGTWTCTAAT-3ʹ). A synthetic *E. coli* 16S rRNA gene sequence in a pUC-like cloning vector (pMA plasmid; GeneArt, ThermoFisher Scientific) was used as a standard. PCR reactions were set up in each well of a 96-well plate using LightCycler 480 SYBR Green I Master Mix. Each sample was run in triplicate and standards in duplicate on a LightCycler 480 Instrument II (Roche). The qPCR conditions were as follows: pre-incubation at 95 °C for 3 min and 45 cycles of denaturation 95 °C for 30 s, annealing at 54 °C for 30 s, and extension at 72 °C for 24 s. 16S rRNA gene copy numbers were calculated based on a standard curve constructed by plotting average C_p_ values of a serial dilution of the plasmid-borne standard against their copy numbers.

### Metagenome sequencing

Metagenomes were analysed from 24 biologically independent samples, specifically three biocrust and three topsoil samples from each of the climatic zones. Samples for metagenomic analysis were chosen based on the community composition of a previous 16S rRNA gene soil survey [30]; PCoA plots of Bray-Curtis dissimilarity were visually inspected and three samples were chosen based on the maximum differences in community composition within each zone (Table S1). Metagenomic shotgun libraries were prepared for 12 biocrust samples using the Nextera XT DNA Sample Preparation Kit (Illumina Inc., San Diego, CA, USA). Sequencing was performed at the Australian Centre for Ecogenomics on a NextSeq500 (Illumina) platform with 2 × 150 bp High Output run chemistry, yielding a total of 283,218,293 paired and 17,363,732 unpaired reads. Metagenomic sequencing of the 12 topsoil samples was previously described [30] and the metagenomes are deposited under BioProject PRJNA642232. For both the new biocrust and existing topsoil metagenomes, raw sequence reads were stripped of adapter and barcode sequences, then contaminating PhiX sequences were identified and removed using the BBDuk function of BBTools v. 36.92 (https://sourceforge.net/projects/bbmap/) with default parameters. Read pairs were then quality trimmed on both ends using BBDuk with Q > 20. After quality filtering and trimming, 96.93% and 96.03% were retained for biocrusts and topsoils respectively. Read counts for the negative controls were 6547 (extraction control) and 1360 (library preparation control) [82].

### Metagenomic community profiling

To profile bacterial, archaeal, and eukaryotic community composition based on the metagenomes, reads encoding 16S rRNA and 18S rRNA genes were retrieved and assigned using PhyloFlash v.3.0 [83]. Given large discrepancies in the taxonomic rank assignment between prokaryotic and eukaryotic taxa, the PhyloFlash analysis was repeated twice. First, the script *phyloFlash.pl* was given a -taxlevel 6 flag for bacteria/archaea specifying a taxonomic assignment down to genus level, followed by a -taxlevel 19 flag for eukaryotes to capture all taxonomic ranks for this domain. Retrieved sequences were then clustered at 97% into OTUs and mapped to the SILVA database (release 138) [81], yielding 4302 bacterial/archaeal OTUs and 5107 eukaryotic OTUs. Of the retrieved gene sequences, 288,788 (94%) mapped to the bacteria/archaeal OTUs and 17,209 (5.7%) mapped to the eukaryotic OTUs. Community composition of bacteria and archaea were also profiled by retrieving single-copy ribosomal marker genes from the biocrust and topsoil metagenomes using default settings of SingleM v.0.12.1 [84]. The single copy marker gene *rplB* was selected for downstream analysis as it was previously identified as a robust means of distinguishing between both closely and distantly related genomes [85]. Sequences were then clustered *de novo* by SingleM into operational taxonomic units (OTUs) using a sequence identity threshold of 97% and mapped to the Genome Taxonomy Database (GTDB r86) [86], yielding a total of 13,996 OTUs. No taxonomic marker sequences were detected in the metagenomes of the negative controls.

### Biodiversity analysis

Alpha and beta diversity were calculated using phyloseq [87] and R package VEGAN [88]. They were profiled based on the 16S rRNA gene amplicon sequences and metagenomic 16S rRNA, 18S rRNA gene, and *rplB* reads. To account for differences in read counts between samples (Fig. S1), reads were normalised to 25,000 for 16S rRNA gene amplicons and 8580, 135, and 584 for metagenomic 16S rRNA gene, 18S rRNA gene, and *rplB* reads, respectively. Observed richness and abundance-based estimated richness (Chao1) were calculated using the *estimate_richness* function in phyloseq. Beta diversity (Bray-Curtis dissimilarity) was calculated and visualised using a multidimensional scaling ordination (PCoA). Permutational analysis of variance (PERMANOVA) was performed to test for significant differences in community structure between soil types and climatic zones, and beta dispersion tests (PERMDISP) were used to ascertain if observed differences were influenced by dispersion. Similarity percentage analysis (SIMPER) [89] was used to further determine which taxa at the class level contribute to compositional changes. The average contribution to compositional change between climatic zones, one standard deviation, and cumulative sum was calculated at the class level using default parameters with 999 permutations.

### Metagenome assembly and binning

Raw reads were quality-controlled using Read_QC module in the metaWRAP pipeline [90]. For each sample, the quality-controlled metagenome was assembled using MEGAHIT v1.1.3 [91] (default parameters). In addition, they were co-assembled using MEGAHIT v1.2.9 (--k-min 31 --kmin-1pass). The resulting assemblies were binned using the binning module within the metaWRAP pipeline (--metabat2 --maxbin2 –concoct for individual assembly; --metabat2 for co-assembly). For each of the 24 individual assemblies, the three bin sets were then consolidated into a final bin set with the bin_refinement module of metaWRAP. The final bin sets from both individual assemblies and co-assembly were aggregated and dereplicated using dRep v2.5.4 [92] (-comp 50 -con 10 options). One dereplicated MAG (bin004) was retained from a previous assembly and added to the final MAG set. For this MAG, trimmed reads were assembled using SPAdes in metagenomic mode [93], using independent assemblies for each sample. Contigs shorter than 1 kbp in length were removed from the assembly using seqmagick (https://github.com/fhcrc/seqmagick/). Reads were then mapped to the assembly using BamM (http://ecogenomics.github.io/BamM/) to create coverage profiles. Contigs assembled from each sample were then binned according to coverage and composition using GroopM2 [94], MetaBAT2 [95], and MaxBin [96], and a representative set of non-redundant bins selected using DAS_Tool [97]. The full set of all bins (i.e., those from all assemblies) were pooled and dereplicated using dRep [92]. For the dereplicated bin set, RefineM [98] was used to identify outlier contigs which may have been incorrectly binned, and these were removed from their respective bins using seqmagick. For all MAGs, completeness and contamination estimates were calculated using CheckM [99], and taxonomy assigned to the refined, dereplicated bins using the GTDB-Tk software [100].

### Metagenome annotation

To estimate the metabolic capability of the soil communities, metagenomic short reads and metagenome-assembled genomes were searched against 51 custom protein databases of representative metabolic marker genes using DIAMOND v.0.9.31 [101]. The custom databases have been previously described [73, 102] (available at 10.26180/c.5230745) and encompass major metabolic marker genes associated with organic and inorganic electron donor utilization, aerobic and anaerobic electron acceptor utilization, photosynthesis and light harvesting, carbon fixation, and nitrogen fixation. For metagenomic short reads, quality-filtered and trimmed metagenomic forward reads (mean length 140 bp; minimum length 100 bp) were searched using the DIAMOND *blastx* algorithm [103] with a query coverage >80% and gene-specific identity thresholds (60% for NxrA, AmoA, CoxL, group 4 [NiFe]-hydrogenases, [FeFe]-hydrogenases, 70% for PsbA and IsoA; 75% for HbsT; 80% for PsaA; 40% for energy-converting rhodopsins; 50% for all other genes). Read counts were converted to reads per kilobase million (RPKM) to normalise to gene length and metagenome size. To estimate the percentage of community members encoding each gene, marker gene abundance was calculated relative to the set of 14 universal single-copy ribosomal genes packaged with SingleM (DIAMOND blastx, query coverage of 80% and bitscore threshold of 40) [73]. Specifically, reads aligning to the single-copy ribosomal genes were converted to RPKM and averaged across the 14 genes to produce one number per sample representing the abundance of a single-copy gene carried by 100% of community members. The RPKM of each metabolic gene was then divided by this number to find the estimated percentage of the community with the gene, assuming one copy per genome. For MAGs and unbinned contigs, open reading frames (ORFs) were predicted using Prodigal v2.6.3 [104]. The obtained ORFs were searched against the 51 custom databases using the DIAMOND *blastp* algorithm with query coverage >80% and gene-specific identity thresholds as previously described [73, 102]. No metabolic marker sequences were detected in the metagenomes of the negative controls.

### Phylogenetic analysis

Maximum-likelihood phylogenetic trees were constructed to visualize the evolutionary relationships of unbinned and binned contigs of the catalytic subunits of [NiFe]-hydrogenase and RuBisCO (RbcL) compared to reference sequences. Retrieved sequences were aligned to custom databases using ClustalW in MEGA7 [105]. For phylogenetic tree construction, initial trees for the heuristic search were obtained automatically by applying Neighbour-Join and BioNJ algorithms to a matrix of pairwise distances estimated using a JTT model, and then selecting the topology with superior log likelihood value. All residues were used and trees were bootstrapped with 50 replicates.

### Soil wetting

We simulated rainfall conditions to determine the effects of soil moisture on H_2_ oxidation and photosynthetic carbon fixation rates. To do this, we used a custom Perspex collar fitted with a water-draining stainless steel woven mesh (0.17 mm) and a water-catching tray (Fig. 1b). Collars were sterilised using ethanol. Topsoil and biocrust samples of 5 g were placed in the centre of the mesh surface. Soils were then watered until fully saturated by repeated addition of 1 mL MilliQ water. Once fully saturated, each collar was sealed at the top using cling film to avoid evaporation and left to drain for 24 h in the dark.

### Gas chromatography

*Ex situ* rates of atmospheric H_2_ oxidation by the 48 biologically independent biocrusts and topsoils were measured by gas chromatography. Samples of 5 g were suspended in 120 mL serum vials and left to equilibrate with ambient air (12 h). Vials were then sealed with a butyl rubber septum and amended with H_2_ (*via* 1% v/v H_2_ in N_2_ gas cylinder, 99.999% pure) to achieve headspace mixing ratios of ~10 ppmv. Sampling commenced immediately after sealing the vial to measure the initial uptake rates. Headspace H_2_ mixing ratios in samples were measured by gas chromatography using a pulsed discharge helium ionization detector (model TGA-6791-W-4U-2, Valco Instruments Company Inc.) as previously described [46]. Rates of uptake were measured for all 48 biocrust and 48 topsoil subsamples under both dry and wet conditions. Heat killed samples (two autoclave cycles at 120 °C) and blank measurements (empty serum vials) were used as controls to confirm that oxidation occurred due to biotic processes. Mixing ratios of H_2_ in each sample were regularly calibrated against ultra-pure gas standards of known concentrations.

### ^14^C isotope labelling

A radiolabelled carbon dioxide (^14^CO_2_) incubation assay was used to measure the capacity of communities within wetted biocrusts and topsoils to mediate three processes: (i) dark CO_2_ assimilation, (ii) hydrogenotrophic CO_2_ fixation, and (iii) photosynthetic CO_2_ fixation. The six biological replicates were pooled for each climatic zone and technical triplicates of 0.25 g were weighed and transferred to 4 ml glass vials sealed with rubber septum lids. For each replicate, a heat killed control (two autoclave cycles at 120 °C) was used. Gaseous ^14^CO_2_ (1% v/v) gas stocks were generated by adding 75 µl of sodium bicarbonate solution (NaH^14^CO_3_, Perkin Elmer, 53.1 mCi nmol^−1^) to 75 µl of 10% hydrochloric acid (HCl) solution inside a 4 ml glass vial, which was sealed with a rubber septum lid and incubated for two hours at room temperature. 160 µl of ^14^CO_2_ gas (1% v/v) was added to each biocrust or topsoil sample using a 1 ml gas tight syringe (SGE Analytical Science), obtaining initial headspace mixing ratios of 400 ppmv ^14^CO_2_. For the first experiment, treatment groups were incubated under either light (40 µmol photons m^−2^ s^−1^ under constant illumination) or dark conditions (covered in aluminium foil) for 96 hours at ~20 °C. For the second experiment, treatment groups were incubated either with or without additional H_2_ gas; H_2_ gas (1% v/v, AirLiquide) was added to half of the sampling cohort to obtain mixing ratios of 100 ppmv H_2_. In both experiments, to remove any unfixed ^14^CO_2_, incubated soils were transferred to 12 ml scintillation vials and suspended in 2 ml of 1 M HCl and left to dry under a heating element at 45–50 °C. Once dried, 10 ml of scintillation cocktail (EcoLume) was added and radioisotope analysis was carried out using a liquid scintillation spectrometer (Tri-Carb 2810 TR, Perkin Elmer precisely) operating at ~95% efficiency. Background luminescence and chemiluminescence were corrected through internal calibration standards.

### Statistical analysis

All statistical analysis was carried out in R v3.5.3. Data manipulation, summaries, and visualization were carried out using the package Tidyverse [106]. Generalised linear models (GLM) were used to test for significant differences in richness and abundance along the aridity gradient. The GLM was fitted with a negative binomial distribution, which was chosen for downstream analysis after comparing optimal model fits against the residual plots of Poisson and quasi-Poisson distributions [107]. For H_2_ oxidation and ^14^C-CO_2_ fixation rates, normality of the data was assessed using the Shapiro-Wilk test. Kruskall-Wallis tests were used to test for significant differences in biogeochemical activities along the aridity gradient. Dunn tests with a Benjamini-Hochberg correction were used for post-hoc testing to identify significant pairs. To identify physicochemical predictors of H_2_ oxidation rates and gene abundance, a Pearson’s correlation matrix was calculated from 21 separate soil chemical parameters previously described [30]. Co-linear predictors (*R*^2^ > 0.8) were removed to determine the final subset of six predictor variables using a variance inflation cut-off of <5. Linear models were then used to estimate the variance in the biochemical and abundance data explained by environmental predictors.

## Results

### Biocrusts and topsoils harbour diverse microbial communities that are structured by aridity

We analysed biocrusts (0–2 cm depth) and previously reported underlying topsoils (2–10 cm depth) [30] sampled along a 160 km latitudinal aridity gradient in Israel (Fig. 1a, Table S1). Soil physicochemical analysis confirmed expected environmental variation along the transect, with soil water and organic carbon content respectively dropping from (average percentage ± one standard deviation) 5.11 ± 2.58% and 3.58 ± 0.62% in the sub-humid northern zones to 1.08 ± 0.30% and 0.10 ± 0.02% in the hyper-arid southern zones [30]. Abundance, richness, and composition of the sampled microbial communities were profiled using quantitative PCR, amplicon sequencing, and metagenomic sequencing respectively (Fig. 1, Table S1). Bacterial and archaeal abundance (16S rRNA copy number based on qPCR) significantly decreased with aridity, with a 61-fold higher copy number in sub-humid compared to hyper-arid samples (Fig. 1f, Table S2). In contrast, estimated richness (Chao1 based on 16S rRNA gene sequencing) between sites was variable and significantly declined with aridity for biocrust but not soil samples (Fig. 1d, Table S2). Beta diversity analysis (Bray-Curtis dissimilarity) revealed that community composition was significantly differentiated between climatic zones (F = 6.1, *p* < 0.001), as well as between biocrusts and topsoils (F = 6.2, *p* < 0.001) (Fig. 1e, Table S2). Metagenomic sequencing also indicated that aridity influences richness and composition (Fig. S2), though richness estimates were more reliable for amplicon sequencing due to higher sequencing coverage of the taxonomic marker gene (Fig. S1). Altogether, these analyses suggest that aridity significantly influences the composition of microbial communities in desert ecosystems, though rich communities can exist even in the driest sites.

**Fig. 2:**
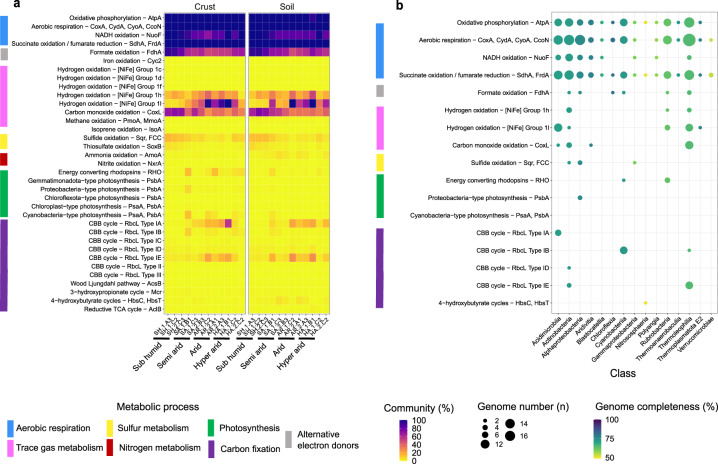
Distribution of energy and carbon acquisition genes in biocrust and topsoil microbial communities sampled along the aridity gradient. **a** Heatmap showing the abundance of metabolic marker genes in the metagenomic short reads. The percentage of the total community predicted to encode at least one of each gene for a process is shown, based on normalization to single-copy marker genes. **b** Dot plot showing the metabolic potential of the 68 metagenome-assembled genomes (MAGs). The size of each point represents the number of genomes in each phylum that encode the gene of interest and the shading represents the average genome completeness.

Bacterial and archaeal community composition was primarily profiled from 16S rRNA gene reads retrieved from the metagenomes (Fig. 1c). At the phylum level, community composition of the samples was similar to that described in most other desert soils [11, 20, 28]. Most sequences affiliated with Actinobacteriota across the gradient (average 52% relative abundance), particularly classes Thermoleophilia, Actinobacteria, Acidimicrobiia, and Rubrobacteria, with significant proportions of Proteobacteria (13%), Chloroflexota (8.2%), Gemmatimonadota (4.8%), and Acidobacteriota (3.7%) also detected (Fig. 1c, Table S3). Most of these bacteria are likely to be aerobic organoheterotrophs, but may also harbour hidden metabolic flexibility. Of the microbial classes detected, Thermoleophilia was the most significantly enriched with increasing aridity and in topsoils compared to crusts (Table S3, Fig. S3). Archaea were much less abundant (0.2%) and were primarily affiliated with putatively ammonia-oxidizing Nitrososphaeraceae [108]. Cyanobacteria were fourfold more abundant in biocrusts (4.2%) compared to topsoils (0.9%) and reached abundances exceeding 5% in five biocrust samples (most notably SA.1.B1 in the semi-arid zone, 19.5%) (Fig. 1d, Table S3). 58% of cyanobacterial sequences affiliated with the cosmopolitan biocrust genus *Microcoleus* [13, 18], with *Coleofasciculus*, *Leptolyngbya*, *Nostoc*, and *Aliterella* also abundant in specific samples (Fig. S4, Table S3). Cyanobacteriia was the class most significantly differentiated by both aridity (peaking in the semi-arid and arid zones) and sample type (enriched in biocrusts versus topsoil) (Fig. S3, Table S3). Concordant observations of an Actinobacteriota-dominated microbial community were also made through 16S rRNA gene amplicon sequencing and assignment of metagenomic single-copy ribosomal protein (*rplB*) reads (Fig. S2, Tables S1, S3). However, these methods seemingly overestimated and underestimated cyanobacterial abundance respectively in a manner inconsistent with the relative abundance of cyanobacterial metabolic marker genes in metagenomes (Fig. 2).

We also profiled eukaryotic community composition based on metagenomic 18S rRNA gene reads (Fig. S5, Tables S1, S3). Eukaryotes accounted for 5.7% of the rRNA small subunit gene reads retrieved from the metagenomes. Fungi (primarily Ascomycota), Animalia (various invertebrates), and protists (including Ciliophora, Cercozoa, and Euglenozoa) were widespread in the biocrusts and topsoils from each zone. Various photoautotrophs were also detected, including vascular plants (Tracheophyta), mosses (Bryophyta), and green algae (Chlorophyta), which were respectively most abundant in the biocrusts of the sub-humid, semi-arid, and arid/hyper-arid zones (Fig. S5). The relatively low abundance of Cyanobacteria and Chlorophyta in the sub-humid zone may reflect that, in contrast to the other climatic zones that contained minimal vegetation, this zone is a shrubland where plants likely dominate primary production (Fig. 1a). Altogether, this lends support for a shift in dominance from multicellular to unicellular primary producers across the aridity gradient.

### Genes encoding chemosynthetic and photosynthetic enzymes are differentially distributed along the aridity gradient

We performed homology-based searches of the metagenomes against a curated database of 51 metabolic marker genes [102] to determine the abundance of different energy and carbon acquisition processes across the desert samples (Table S4). Consistent with our inferences from the community composition profile, most bacteria were predicted to be capable of aerobic organoheterotrophy, with reads encoding NADH dehydrogenases, succinate dehydrogenases, and terminal oxidases particularly abundant. However, a large proportion of the community were also predicted to oxidize atmospheric trace gases, with an average of 73% and 48% of community members predicted to encode the catalytic subunits of uptake (group 1 and 2) [NiFe]-hydrogenases and form I carbon monoxide dehydrogenases respectively across the aridity gradient (Fig. 2a). The abundance of these genes remained relatively consistent between biocrusts and topsoils, suggesting that the potential for these processes is dominant throughout the upper 10 cm of these soils (Table S4). In contrast, fewer microorganisms were predicted to oxidize sulfide (7.9%), thiosulfate (2.2%), ammonia (1.6%), nitrite (0.23%), methane (0.28%), or iron (0.03%) (Table S4, Fig. 2a). A moderate proportion of the community were also capable of using nitrate as an electron acceptor (12%), whereas other genes for anaerobic respiration or fermentation genes were negligible (Fig. S6, Table S4). Marker genes for various carbon fixation pathways were also detected, with the most abundant being RuBisCO (24%) (Fig. 2a, Table S4).

Of the 51 genes screened, those encoding hydrogenases and RuBisCO exhibited the steepest variations in relative abundance across the aridity gradient. All four hydrogenase subgroups known to support atmospheric H_2_ oxidation were detected, namely the group 1h, 1l, 2a, and 1f [NiFe]-hydrogenases (Fig. 2, Table S4), with the first two most abundant. The group 1h enzymes, which are the main clade thought to mediate atmospheric H_2_ oxidation in global soils [42, 43, 57], were abundant in all climatic zones and encoded by an average of 22% of community members. However, in common with recent observations made in terrestrial Antarctic deserts [73], the recently discovered group 1l [NiFe]-hydrogenase was the most abundant lineage in oligotrophic soils. The relative abundance of this lineage increased between climatic zones, with most bacteria in the arid and hyper-arid zones predicted to encode it (sub-humid 6.3%, semi-arid 32%, arid 82%, hyper-arid 83%) (Fig. 2a, Table S4). Consistently, linear models showed that the abundance of this hydrogenase in topsoils was significantly negatively related with soil water content (*R*^2^ = 0.52, *p* = 0.0011) and organic carbon content (*R*^2^ = 0.41, *p* = 0.0004), with similar but weaker trends observed for biocrusts (Fig. S7). There was also a concomitant increase in the relative abundance of two RuBisCO lineages, type IA and type IE RuBisCO (from 0.62% and 3.5% in the sub-humid zone to 17% and 12% in the hyper-arid zone, respectively) (Fig. 2a, Table S4), with most hits closely related to actinobacterial lineages known to encode high-affinity hydrogenases [70, 109]. The hydrogenase and RuBisCO results together suggest that, in common with Antarctic desert soils [55], Negev desert bacteria also use electrons derived from atmospheric H_2_ to catalyse CO_2_ fixation. The abundance of CO dehydrogenases showed the opposite trend, declining from 70% in the sub-humid zones to 35% in the hyper-arid zone (Fig. 2a, Table S4), in agreement with previous studies showing CO oxidation rates are positively correlated with soil organic content [110, 111].

Genes encoding photosystems and RuBisCO lineages associated with oxygenic photosynthesis were also differentially distributed across metagenomes. Most of the short reads predicted to encode these enzymes were more closely related to reference sequences of photosynthetic Cyanobacteria than eukaryotes (Table S4) type IB RuBisCO, a lineage specific to Cyanobacteria and photoautotrophic eukaryotes [112], was enriched in biocrusts (2.5%) compared to topsoils (1.1%) (Fig. 2a). Genes encoding this enzyme were most abundant in specific biocrust samples from the semi-arid, arid, and hyper-arid zones (Fig. 2a, Table S4), corresponding to the sites with a high relative abundance of Cyanobacteria (Fig. 1c), though variations did not strongly correlate with measured physicochemical variables (Fig. S7). Together with the community analysis, these results highlight that there is some potential for photosynthesis even in arid and hyper-arid regions, but photoautotrophs have a lower and more variable distribution than hydrogenotrophs.

### Three actinobacterial classes encode diverse uptake hydrogenase and RuBisCO enzymes across the aridity gradient

To gain a more detailed perspective of the mediators of photosynthesis and chemosynthesis, we individually and collectively assembled and binned the 24 metagenomes. This resulted in the recovery of 68 medium- and high-quality [113] metagenome-assembled genomes (MAGs) from seven bacterial and two archaeal phyla (Fig. 2b, Table S5). In line with the community composition (Fig. 1c; Fig. S2), 69% of the MAGs affiliated with the Actinobacteriota classes Thermoleophilia, Actinobacteria, Acidimicrobiia, and Rubrobacteria and the recently named class *Ca*. Aridivitia [73]. Six cyanobacterial genomes were also constructed, including from the three genera most abundant in the Negev biocrusts, *Microcoleus*, *Coleofasciculus*, and *Leptolyngbya* (Table S5, Fig. S4). As expected, MAG annotations suggested that Actinobacteriota and most other phyla encode genes for aerobic organotrophic respiration, whereas the dominant Cyanobacteria are oxygenic photoautotrophs. The metabolic annotations suggest many Actinobacteriota also have the capacity to conserve energy and fix carbon through trace gas oxidation; uptake hydrogenases, CO dehydrogenases, and RuBisCO were encoded by 46%, 26%, and 30% of MAGs from this phylum respectively (66%, 38%, and 44% when normalized to MAG completeness) (Fig. 2b, Table S6). In a further indication of the hidden metabolic versatility of desert Actinobacteriota, we recovered MAGs of three *Rubrobacter* lineages capable of light harvesting via bacteriorhodopsin and a *Mycobacterium* lineage encoding a key enzyme for aerobic sulfide oxidation (sulfide-quinone oxidoreductase) (Fig. 2b, Table S6). This adds to the growing evidence that desert bacteria continually harvest energy from atmospheric, lithic, and solar sources [11, 20, 102, 114].

**Fig. 3:**
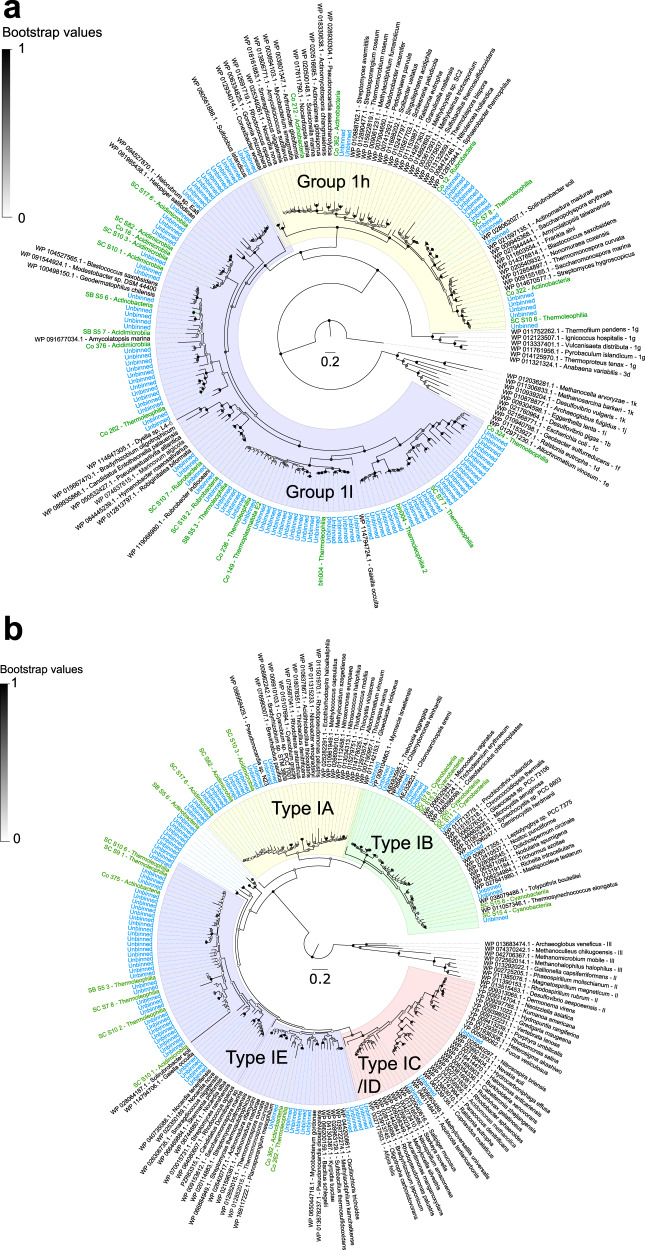
Maximum likelihood radial phylogenetic trees showing sequence diversity and taxonomic distribution of enzymes responsible for H_2_ oxidation and carbon fixation. **a** Phylogenetic tree of [NiFe]-hydrogenase large subunit amino acid sequences, with a focus on the group 1h (HhyL) and 1l (HylL) high-affinity uptake hydrogenases to which most binned and unbinned sequences affiliated with. **b** Phylogenetic tree of RuBisCO large subunit amino acid sequences (RbcL), with a focus on the type IA (Acidimicrobiia-affiliated), type IB (Cyanobacteria-affiliated), and type IE (Actinobacteria- and Thermoleophilia-affiliated) enzymes that most binned and unbinned sequences grouped with. Trees show hits to genome bins (red) and unbinned contigs (blue) relative to reference amino acid sequences (grey) (color figure online).

To gain a more comprehensive understanding of the basis of primary production across the gradient, we constructed maximum-likelihood trees of the catalytic subunit sequences of [NiFe]-hydrogenases (Fig. 3a) and RuBisCO (Fig. 3b), using sequences retrieved from binned and unbinned contigs (Table S7). The hydrogenase tree confirmed the desert microbial communities encoded diverse group 1h and group 1l [NiFe]-hydrogenases, with almost all binned hits affiliating with the four dominant actinobacterial classes (Actinobacteria, Thermoleophilia, Acidimicrobiia, Rubrobacteria) (Fig. 3a). Multiple Thermoleophilia and Actinobacteria MAGs encode the type IE RuBisCO, the key enzyme proposed to mediate atmospheric chemosynthesis on the Antarctic continent [55, 73, 78], whereas the Acidimicrobiia MAGs formed a novel subclade of the type IA RuBisCO (Fig. 3b). In combination, these trees suggest that the three most abundant bacterial classes in the arid and hyper-arid zones (Fig. 1c, Fig. S2) are capable of hydrogenotrophic carbon fixation. Moreover, they suggest that actinobacterial classes have independently evolved the capacity to oxidize atmospheric H_2_ and fix carbon dioxide on several different occasions. As anticipated from the short-read annotations (Fig. 2a), type IB RuBisCO sequences were also detected in the cyanobacterial MAGs (Fig. 3b, Table S5). Several unbinned sequences were also retrieved that are closely related to reference sequences of Chlorophyta (*Myrmecia israeliensis*) and Bryophyta (*Pseudocrossidium* spp.) known to be abundant in Negev desert biocrusts [115, 116] (Fig. 3b). In combination, these findings suggest that hydrogenotrophic Actinobacteriota are of comparable importance as desert primary producers to classical photoautotrophs such as Cyanobacteria.

### Differential activities of chemosynthetic and photosynthetic microorganisms across the aridity gradient

**Fig. 4:**
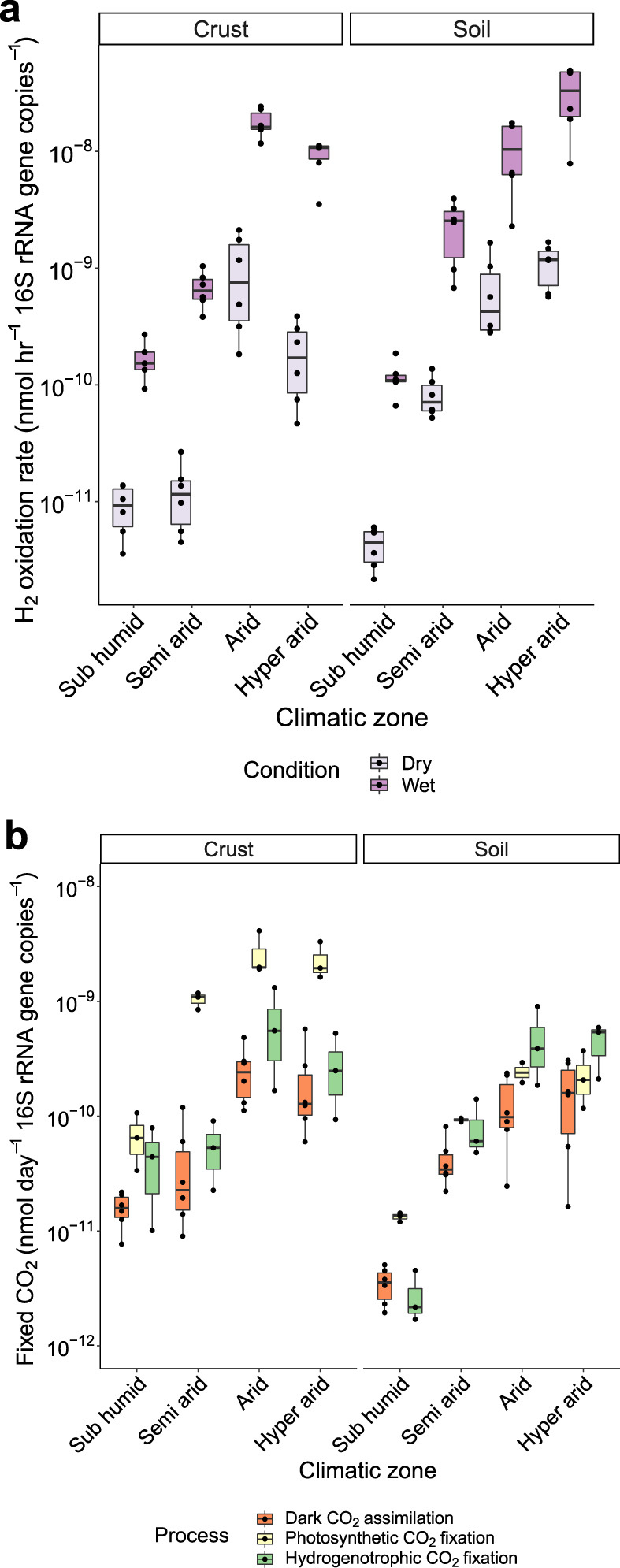
Biogeochemical activity measurements of photosynthetic and hydrogenotrophic processes in biocrusts and topsoils collected along the aridity gradient. **a** Rates of H_2_ oxidation under dry and wet conditions based on gas chromatography. Six biologically independent biocrust and topsoil samples were incubated per climatic zone. **b** Rates of carbon fixation in wet biocrust and topsoil samples based on tracing ^14^C-labelled CO_2_ incorporation. Three processes are shown: dark carbon assimilation (i.e., basal rate of CO_2_ incorporation under dark ambient conditions due to carbon fixation or anaplerotic processes); photosynthetic carbon fixation (i.e., amount of additional CO_2_ fixed under light ambient conditions); and hydrogenotrophic CO_2_ fixation (amount of additional CO_2_ fixed under dark H_2_-enriched conditions). For both biocrusts and topsoils, six biologically independent samples were pooled for each climatic zone and experiments were performed in technical triplicates. For both panels, rates are normalized to 16S rRNA gene copy number as a proxy for biomass (unadjusted rates shown in Fig. S9). Boxplots show median, upper and lower quartile, and minimum and maximum values.

Having confirmed the metabolic potential for atmospheric H_2_ oxidation, we performed microcosm incubations to confirm whether this process was active in the sampled soils. H_2_ oxidation rates were monitored by gas chromatography under dry conditions (soils as collected) and wet conditions (24 h after simulated rainfall event; see Methods). All biocrust and topsoil samples consumed H_2_ to sub-atmospheric levels (Fig. S8, Table S8). In line with recent observations of Australian soils [32, 59], H_2_ oxidation occurred at relatively slow rates under dry conditions and increased an average of 26-fold upon hydration (Fig. S8). Bulk H_2_ oxidation rates increased across the aridity gradient for both the biocrust and topsoil samples (Fig. 4a, Fig. S9). This pattern was particularly evident following hydration and biomass normalization, with cell-specific H_2_ oxidation rates increasing significantly by an average of 142-fold across the aridity gradient for both biocrusts (H = 20.7, *p* = 0.00016) and topsoils (H = 20.0, *p* = 0.00012) (Table S8). Linear models showed that several physicochemical variables were significantly correlated with cell-specific H_2_ oxidation rates, including organic carbon (*R*^2^ = 0.29–0.83, *p* < 0.01), sodium (*R*^2^ = 0.47–0.58, *p* < 0.05), and phosphate (*R*^2^ = 0.74–0.86, *p* < 0.001) content (Fig. S10), which reflects similar observations made for the relative abundance of the group 1l [NiFe]-hydrogenase (Fig. S7). Thus, in agreement with culture-based observations [42, 43, 45, 53, 68], desert Actinobacteriota are predicted to scavenge atmospheric trace gases as alternative energy sources when starved for preferred organic substrates.

We additionally measured rates of hydrogenotrophic and photosynthetic carbon fixation. To do so, we measured ^14^C-CO_2_ incorporation into biomass under dark ambient conditions (to measure dark fixation and/or anaplerotic assimilation), following light stimulation (to measure photosynthetic fixation), and following H_2_ stimulation (to measure hydrogenotrophic fixation). Based on preliminary studies of native dry soils, CO_2_ incorporation was below detection limits under all conditions (data not shown). Following hydration, we could measure significant dark, photosynthetic, and hydrogenotrophic incorporation in all sampled biocrusts and topsoils (Fig. 4b, Figs. S9, S11). All three processes occurred at slow rates in the sub-humid zone, in line with the low abundance of carbon fixation genes in the metagenomes from these sites (Fig. 2a), which likely reflects that plants rather than microorganisms serve as the main primary producers. As expected, photosynthesis dominated primary production in the pooled biocrusts for each zone, occurring at 11-fold higher cell-specific rates compared to the soil samples (Fig. 4b). Whereas bulk rates of photosynthetic carbon fixation were variable (Fig. S9), cell-specific rates increased with aridity (Fig. 4b), suggesting Cyanobacteria in the semi-arid, arid, and hyper-arid zones are highly responsive to hydration. It should be noted that, as we pooled biocrust and topsoil samples per zone due to the high biomass required for this assay, we were unable to detect the variations in fixation rates between samples in a given zone; these would likely be high for photosynthesis given the patchy distribution of Cyanobacteria in crust samples inferred from the metagenomic analyses of community composition (Fig. 1c) and function (Fig. 2a).

Finally, we confirmed our metagenomic predictions that H_2_ oxidation can drive carbon fixation in this ecosystem. Cell-specific rates of hydrogenotrophic carbon fixation increased with aridity across both the soil and biocrust samples (Fig. 4b). This increase is consistent with the observed enrichment of Actinobacteriota harbouring hydrogenase and RuBisCO genes (Figs. 1, 2). Hydrogenotrophic fixation occurred more slowly than photosynthetic fixation in hydrated biocrusts. However, this process occurred at rapid cell-specific rates that exceeded photosynthetic fixation in both arid and hyper-arid topsoils (Fig. 4b). To our knowledge, this provides the first experimental observation of hydrogenotrophic carbon fixation in hot deserts.

## Discussion

**Fig. 5:**
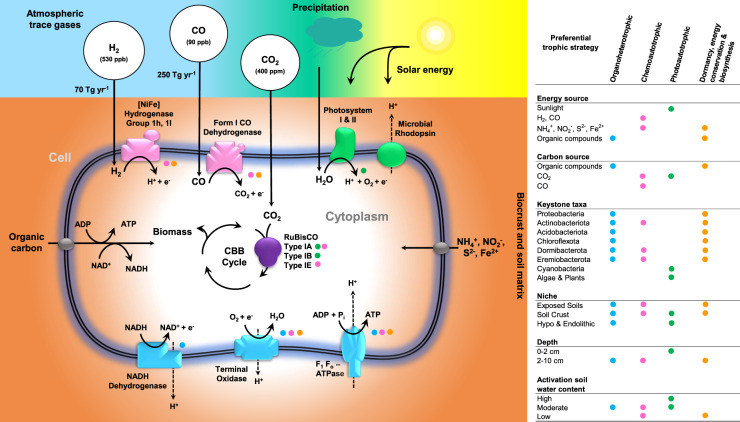
Conceptual infographic of energy conservation and carbon acquisition detected based on desert metagenome-assembled genomes. The key enzymes and taxa mediating organic carbon oxidation, light harvesting, trace gas oxidation, and carbon fixation are shown, as are the niches and conditions that these processes are predicted to be most active in.

In this work, we demonstrate how two key microbial energy conservation strategies, photosynthesis and chemosynthesis, interact with aridity. We summarise the enzymes and microorganisms that mediate these processes, as well as their responses to hydration, in Fig. 5. Extending our previous findings [32, 55, 73], we show that atmospheric H_2_ oxidation is likely to be a dominant metabolic process in hot desert soils. The determinants of atmospheric chemosynthesis are widespread across the aridity gradient and are particularly abundant in the most oligotrophic interior of arid and hyper-arid regions. This is evident from the abundance and diversity of genes associated with aerobic H_2_ oxidation (group 1h and 1l [NiFe]-hydrogenases) and chemosynthetic carbon fixation (type IA and IE RuBisCO genes). The dominant bacterial classes in desert soils, Thermoleophilia, Actinobacteria, and Acidimicrobiia, encode these genes; it is likely these classes independently evolved the capacity to mediate chemosynthetic CO_2_ fixation, given the phylogenetic trees revealed that they encode distinct hydrogenase and RuBisCO subtypes. Biogeochemical studies confirmed that these communities actively consume atmospheric H_2_, though at variable rates. In line with our recent findings [32], activity occurred even under dry conditions and hydration accelerated rates. Biomass-normalised rates greatly increased across the aridity gradient, consistent with culture-based observations that expression and activity of actinobacterial uptake hydrogenases are highest during severe organic carbon starvation [42, 51, 68]. Moreover, we observed rapid hydrogenotrophic carbon fixation in wetted but not dry biocrusts and topsoils, with rates exceeding photosynthetic fixation in the arid and hyper-arid soils. Altogether, this provides the first experimental confirmation that hydrogenotrophic carbon fixation is a major process in hot desert soils and adds to growing evidence that atmospheric H_2_ oxidation enhances survival of the dominant bacteria in oligotrophic ecosystems.

Metagenomic and biogeochemical measurements nevertheless confirmed that oxygenic photosynthesis is a key primary production process across the aridity gradient. In line with expectations [9, 117], various Cyanobacteria were detected such as the keystone crust-forming taxon *Microcoleus*, with their abundance peaking in several semi-arid and arid biocrust samples. Correlating with their presence were genes supporting the light reactions (photosystems) and dark reactions (type IB RuBisCO) of photosynthesis. However, radiolabelling studies confirmed that photosynthetic processes are virtually absent under dry conditions, in contrast to the extensive oxidation of atmospheric H_2_. Measurable carbon fixation was only observed once the electron donor water is introduced, with activity peaking in the semi-arid and arid biocrusts where Cyanobacteria are most abundant. This agrees with previous work showing that simulated rainfall greatly stimulates activities of photoautotrophs in biocrusts and topsoils alike [16, 18]. In the Negev desert, the moisture required for photoautotroph activity is provided on a regular basis by dewfall and more occasionally from rainfall [17, 118]. However, any photosynthesis is likely to be transient. Studies on the cyanobacterium *Leptolyngbya ohadii* suggest that photosynthetic activity is only activated for a short period following morning dewfall, before shutting down due to extreme light intensity, temperature, and desiccation [119, 120]. Similarly, studies on cyanobacterial biocrusts suggest that photosystem II is only activated by heavy fog events, which account for ~5% of the total annual dewfall events in the region [16, 18]. Thus, we conclude that photoautotroph abundance and activity is often high in the Negev desert, though is variable across spatial and temporal scales.

These findings on chemosynthetic and photosynthetic bacteria in the Negev desert have broader ramifications for understanding primary production in desert ecosystems. Cyanobacteria and photoautotrophic eukaryotes are generally thought to be the dominant primary producers in desert ecosystems, which supply organic carbon to organoheterotrophs. Contrary to this paradigm, here we provide metagenomic and biogeochemical evidence that multiple primary producers operate in Negev biocrusts and topsoils. Hydrogenotrophic Actinobacteriota are consistently more abundant than photoautotrophs. Reflecting this, the primarily Actinobacteriota-affiliated type IA and type IE RuBisCO are on average fivefold more abundant across the samples than the primarily Cyanobacteria-affiliated type IB RuBisCO. Moreover, contrary to the long-held assumption that these communities are only active when wet [13, 117], Actinobacteriota appear to mediate rapid H_2_ oxidation in dry conditions even when photoautotrophs are inferred to be inactive. They additionally mediate carbon fixation when hydrated, in common with photoautotrophs, though the highest biomass-normalised rates occurred in the driest soils. It should be noted that, with the exception of one sample from the semi-arid zone, the abundance of the sampled cyanobacterial community is lower than in many previously reported biocrusts [121–123]. This reflects that the dry loess soils of the Negev arid and hyper-arid zones are generally covered by biocrusts that are relatively thin and lightly hued (Fig. 1b) compared to those in semi-arid regions such as the Colorado Plateau. Nevertheless, Actinobacteriota are generally abundant in desert biocrusts, for example as the dominant taxa in certain Mojave and Tengger biocrusts [124–126]. These findings justify further studies to resolve the relative contributions of photosynthetic and chemosynthetic microorganisms in the establishment, maintenance, and productivity of biocrusts.

Integrating these findings, we propose that metabolic flexibility underlies the consistent dominance of the actinobacterial lineages in desert biocrusts and topsoils. These organoheterotrophs take advantage of transient hydration events, using exudates released by phototrophs and the necromass released through osmotic shock to increase respiration rates and accumulate macromolecular stores [127–133]. Their capacity to also conserve energy and fix carbon through trace gas oxidation independently of organic inputs, however, confers a survival advantage during subsequent desiccation and starvation. While atmospheric H_2_ is likely to be the main energy source sustaining these bacteria in desiccated soils, our metagenomic analysis indicates atmospheric carbon monoxide oxidation, lithic sulfide oxidation, and bacteriorhodopsin-mediated light harvesting can also support specific lineages. Our metagenomic and biogeochemical inferences are well-supported by pure culture studies showing sporulating and non-sporulating actinobacterial species alike can survive carbon starvation by utilising atmospheric trace gases [42, 43, 45, 48, 51, 53]. Moreover, the capacity to use trace gases to fix CO_2_ is likely to enable cells to maintain biomass levels and potentially sustain slow growth. Overall, it can be inferred that trace gas oxidation confers a selective advantage for metabolically flexible organoheterotrophs by providing means of acquiring alternative energy donors to sustain basal energy requirements during dormancy and in some cases a mixotrophic means of acquiring biomass. Given the widespread taxonomic distribution of this metabolism [44–46, 50, 70, 134], it is likely that other bacterial and archaeal lineages in these desert soils also possess hidden metabolic flexibility.

At the landscape scale, chemosynthetic primary production is likely to be an important keystone function supporting microbial biodiversity, especially in arid and hyper-arid soils where photoautotrophs are increasingly sparse. These processes are also predicted to enhance the minimal carbon stocks of these soils and likely represent an underestimated fraction of carbon inputs. However, given the energetic constraints of coupling atmospheric H_2_ oxidation to CO_2_ fixation, this process only supports slow biomass formation and hence oligotrophic conditions prevail in arid and hyper-arid deserts. Further work is required to understand how the interrelated factors of hydration (including precipitation and dewfall), carbon availability, and temperature influence chemosynthetic and photosynthetic activities over different temporal and spatial scales.

## Supplementary information

Supplementary material

Table S1

Table S2

Table S3

Table S4

Table S5

Table S6

Table S7

Table S8

## Data Availability

All metagenomes sequenced for this project are available on the Sequence Read Archive under BioProject accession number PRJNA657906. Metagenome-assembled genomes have been deposited on Figshare (doi 10.6084/m9.figshare.12818810). No custom code was used for this study.
