# Supplementary material for: Chemosynthetic and photosynthetic bacteria contribute differentially to primary production across a steep desert aridity gradient
Source: ISME J. 2021 May 25;15(11):3339–56. doi: 10.1038/s41396-021-01001-0 (PMC8528921; doi:10.1038/s41396-021-01001-0)

Supplementary information for

'Chemosynthetic and photosynthetic bacteria contribute differentially to primary production across a steep desert aridity gradient'

Sean K. Bay * [1,2], David W. Waite[3,4], Xiyang Dong[5], Osnat Gillor[6], Steven L. Chown[2], Philip Hugenholtz[3], Chris Greening * [1,2]

[1] Department of Microbiology, Biomedicine Discovery Institute, Monash University, Clayton, VIC 3800, Australia

[2] School of Biological Sciences, Monash University, Clayton, VIC 3800, Australia

[3] Australian Centre for Ecogenomics, School of Chemistry and Molecular Biosciences, St Lucia, QLD 4072, Australia

[4] School of Biological Sciences, University of Auckland, Auckland 1010, New Zealand

[5] School of Marine Sciences, Sun Yat-Sen University, Zhuhai 519082, China

[6] Zuckerberg Institute for Water Research, Blaustein Institutes for Desert Research, Ben Gurion University of the Negev, Sde Boker, Israel

* Correspondence should be addressed to:

A/Prof Chris Greening (chris.greening@monash.edu)

Dr Sean K. Bay (sean.bay1@monash.edu)

Supplementary Tables

Table S1 (xlsx). Summary of sampling design, sample attributes, and taxa identified through 16S rRNA gene amplicon sequencing and metagenomic profiling of the 16S rRNA gene, 18S rRNA gene, and *rplB* gene.

Table S2 (xlsx). Details and statistics of microbial abundance, alpha diversity, and beta diversity across the aridity gradient.

Table S3 (xlsx). Relative abundance of bacteria and archaea at phylum and class levels in sampled biocrusts and topsoils, and analysis of differences across climatic zones and plots based on SIMPER analysis.

Table S4 (xlsx). Distribution of metabolic genes in metagenomic short reads based on homology-based searches.

Table S5 (xlsx). Assembly statistics and taxonomic classification of metagenome-assembled genomes.

Table S6 (xlsx). Distribution and sequences of metabolic genes in metagenome-assembled genomes.

Table S7 (xlsx). Amino acid sequences of the [NiFe]-hydrogenase and RuBisCO catalytic subunits detected in the metagenome-assembled genomes and unbinned contigs.

Table S8 (xlsx). Rates and statistics of H_2 oxidation and CO_2 fixation measured for biocrusts and topsoils under both dry and wet conditions.

Supplementary Figures

Figure S1. Comparison of rarefaction curves and rarefying thresholds (dotted blue line) for taxonomic profiling based on 16S rRNA gene amplicon sequencing and metagenomic sequencing. Results are shown for **(a)** 16S rRNA gene amplicon sequence variants (ASVs), **(b)** metagenomic *rplB* operational taxonomic units (OTUs; SingleM), **(c)** metagenomic 16S rRNA gene nearest taxonomic units (NTUs, PhyloFlash), and **(d)** metagenomic 18S rRNA gene nearest taxonomic units (NTUs, PhyloFlash).

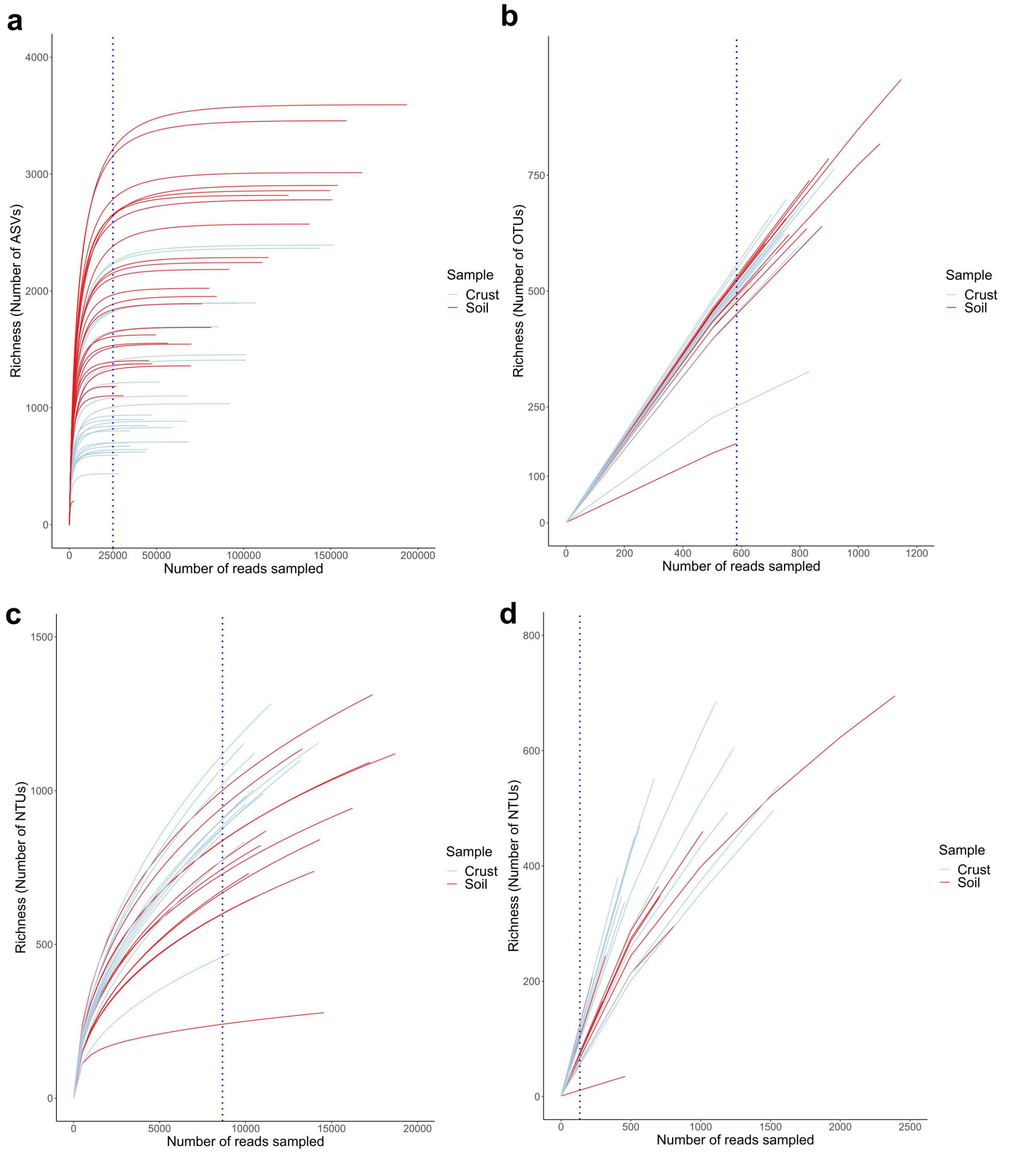

Figure S2. Comparison of estimates of bacterial/archaeal composition, richness, and beta diversity based on amplicon and metagenomic sequencing. Stacked barcharts show class-level relative abundance based on **(a)** 16S rRNA gene amplicon sequencing (n = 48), **(b)** metagenomic 16S rRNA gene sequences (n = 24; as per **Fig. 1c**), and **(c)** metagenomic *rplB* gene sequences (n = 24). Boxplots of estimated richness (Chao1) are shown based on **(d)** metagenomic 16S rRNA gene sequences and **(f)** metagenomic *rplB* gene sequences (equivalent results based on 16S rRNA gene amplicon sequencing shown in **Fig. 1d**). Beta diversity (Bray-Curtis dissimilarity) PCoA ordinations are shown based on **(e)** metagenomic 16S rRNA gene sequences and **(g)** metagenomic *rplB* gene sequences (equivalent results based on 16S rRNA gene amplicon sequencing shown in **Fig. 1e**).

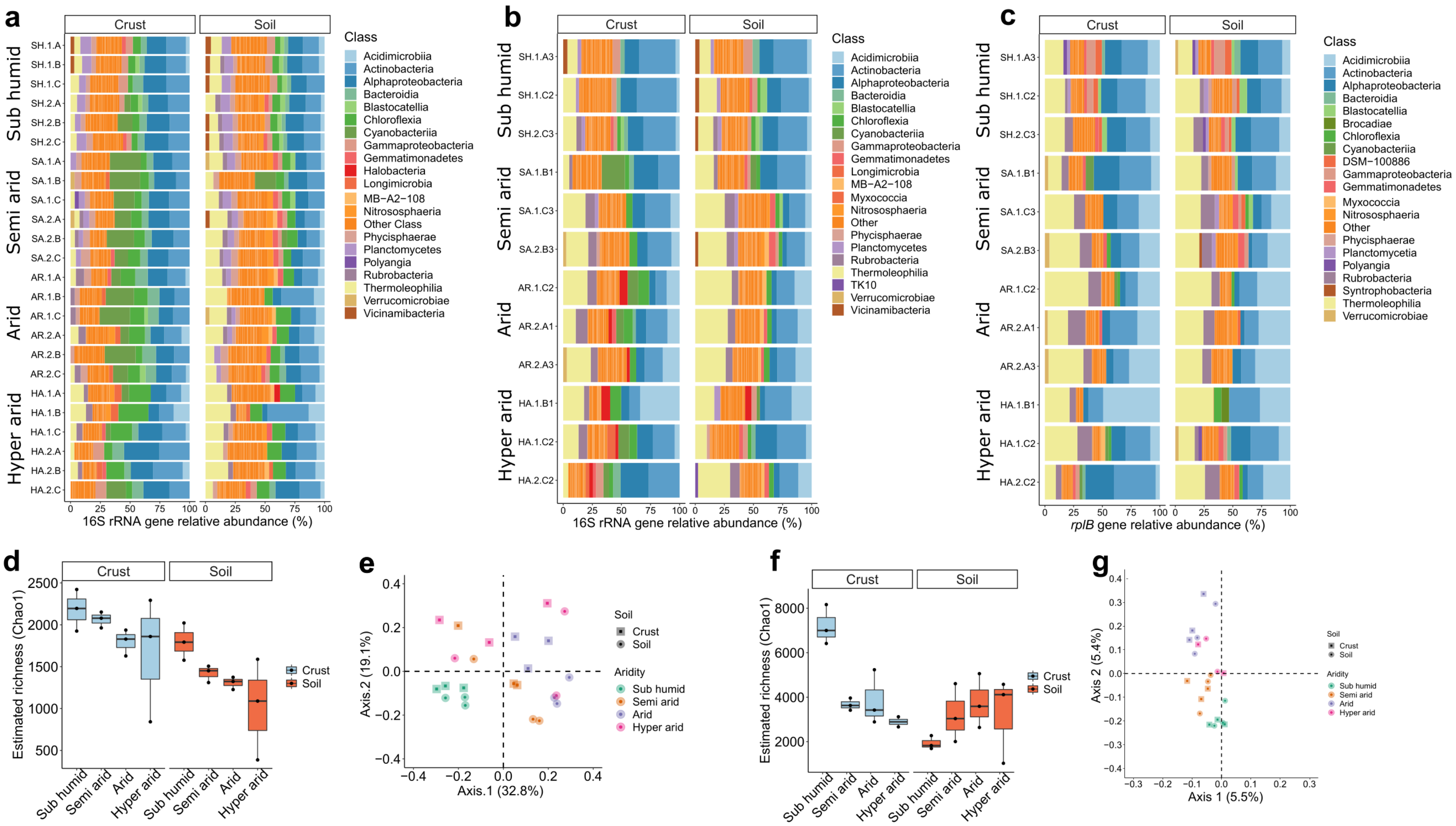

a
Crust
Soil
Sub humid
Semi arid
Arid
Hyper arid
SH.1.A
SH.1.B
SH.1.C
SH.2.A
SH.2.B
SH.2.C
SA.1.A
SA.1.B
SA.1.C
SA.2.A
SA.2.B
SA.2.C
AR.1.A
AR.1.B
AR.1.C
AR.2.A
AR.2.B
AR.2.C
HA.1.A
HA.1.B
HA.1.C
HA.2.A
HA.2.B
HA.2.C
16S rRNA gene relative abundance (%)
Class
Acidimicrobiia
Actinobacteria
Alphaproteobacteria
Bacteroidia
Blastocatellia
Chloroflexia
Cyanobacteriia
Gammaproteobacteria
Gemmatimonadetes
Halobacteria
Longimicrobia
MB−A2−108
Nitrososphaeria
Other Class
Phycisphaerae
Planctomycetes
Polyangia
Rubrobacteria
Thermoleophilia
Verrucomicrobiae
Vicinamibacteria
b
Crust
Soil
SH.1.A3
SH.1.C2
SH.2.C3
SA.1.B1
SA.1.C3
SA.2.B3
AR.1.C2
AR.2.A1
AR.2.A3
HA.1.B1
HA.1.C2
HA.2.C2
16S rRNA gene relative abundance (%)
Class
Acidimicrobiia
Actinobacteria
Alphaproteobacteria
Bacteroidia
Blastocatellia
Chloroflexia
Cyanobacteriia
Gammaproteobacteria
Gemmatimonadetes
Longimicrobia
MB−A2−108
Myxococcia
Nitrososphaeria
Other
Phycisphaerae
Planctomycetes
Rubrobacteria
Thermoleophilia
TK10
Verrucomicrobiae
Vicinamibacteria
c
Crust
Soil
rplB gene relative abundance (%)
Class
Acidimicrobiia
Actinobacteria
Alphaproteobacteria
Bacteroidia
Blastocatellia
Brocadiae
Chloroflexia
Cyanobacteriia
DSM−100886
Gammaproteobacteria
Gemmatimonadetes
Myxococcia
Nitrososphaeria
Other
Phycisphaerae
Planctomycetia
Polyangia
Rubrobacteria
Syntrophobacteria
Thermoleophilia
Verrucomicrobiae
d
Crust
Soil
Estimated richness (Chao1)
Sub humid
Semi arid
Arid
Hyper arid
e
Axis.1 (32.8%)
Axis.2 (19.1%)
Soil
Crust
Soil
Aridity
Sub humid
Semi arid
Arid
Hyper arid
f
Crust
Soil
Estimated richness (Chao1)
g
Axis 1 (5.5%)
Axis 2 (5.4%)
Soil
Crust
Soil
Aridity
Sub humid
Semi arid
Arid
Hyper arid

Figure S3. Similarity percentage analysis (SIMPER) of relative contribution of different microbial classes to composition change between climatic zones based on 16S rRNA gene amplicon sequencing. Shown are the top 24 taxa contributing to 80% of community compositional changes between climatic zones. Bars represent mean values for the biocrusts (n = 24; six per zone) and topsoils (n = 24; six per zone). For each value, one standard deviation is shown in black error bars.

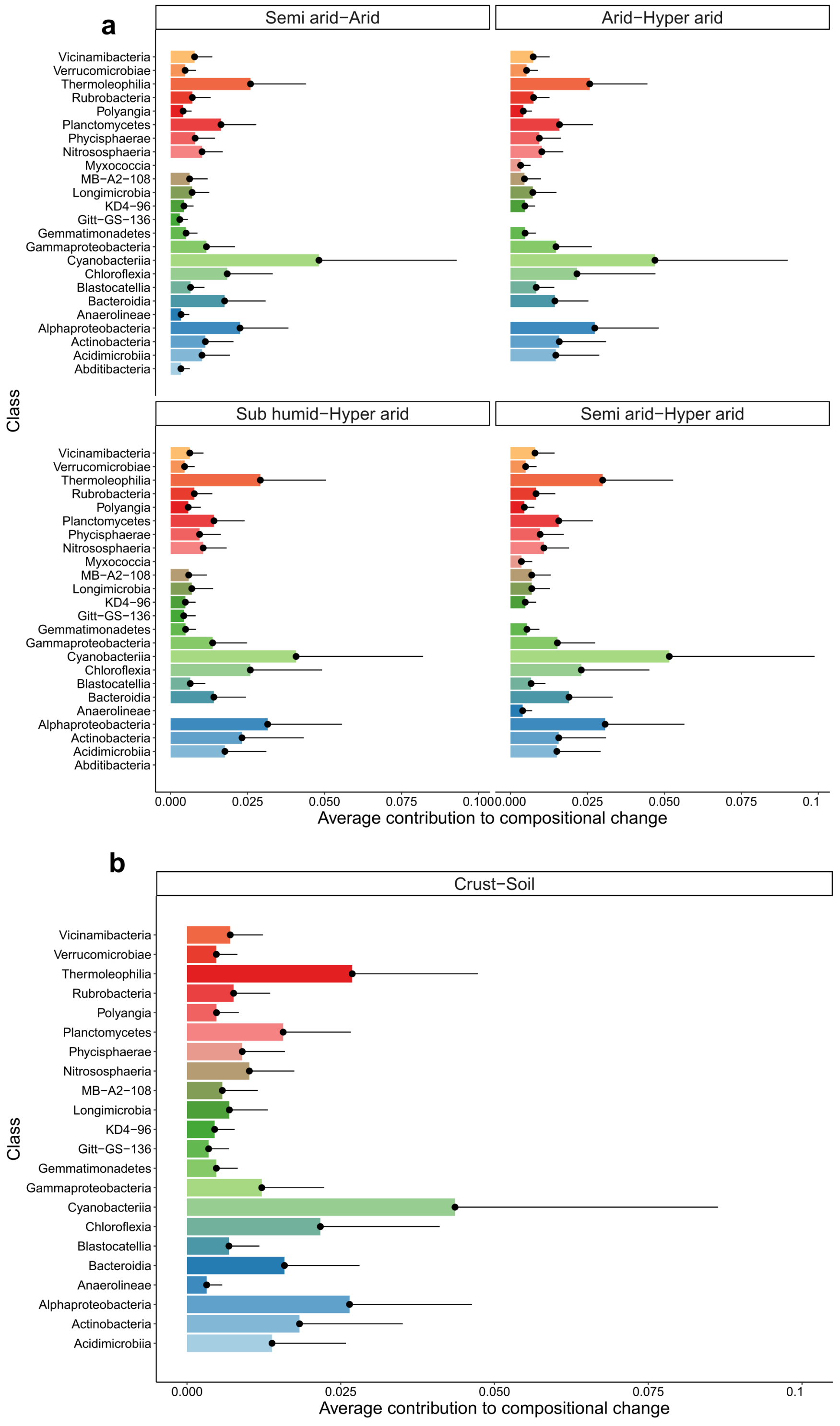

Figure S4. Stacked barchart showing the genus-level abundance of cyanobacterial genera detected along the aridity gradient based on 16S rRNA gene amplicon sequencing. Genera were classified according to the Genome Taxonomy Database (GTDB). Vampirovibrionales and Obscuribacteraceae affiliate with the non-photosynthetic classes, whereas the other genera affiliated with the photosynthetic class Cyanobacteriia.

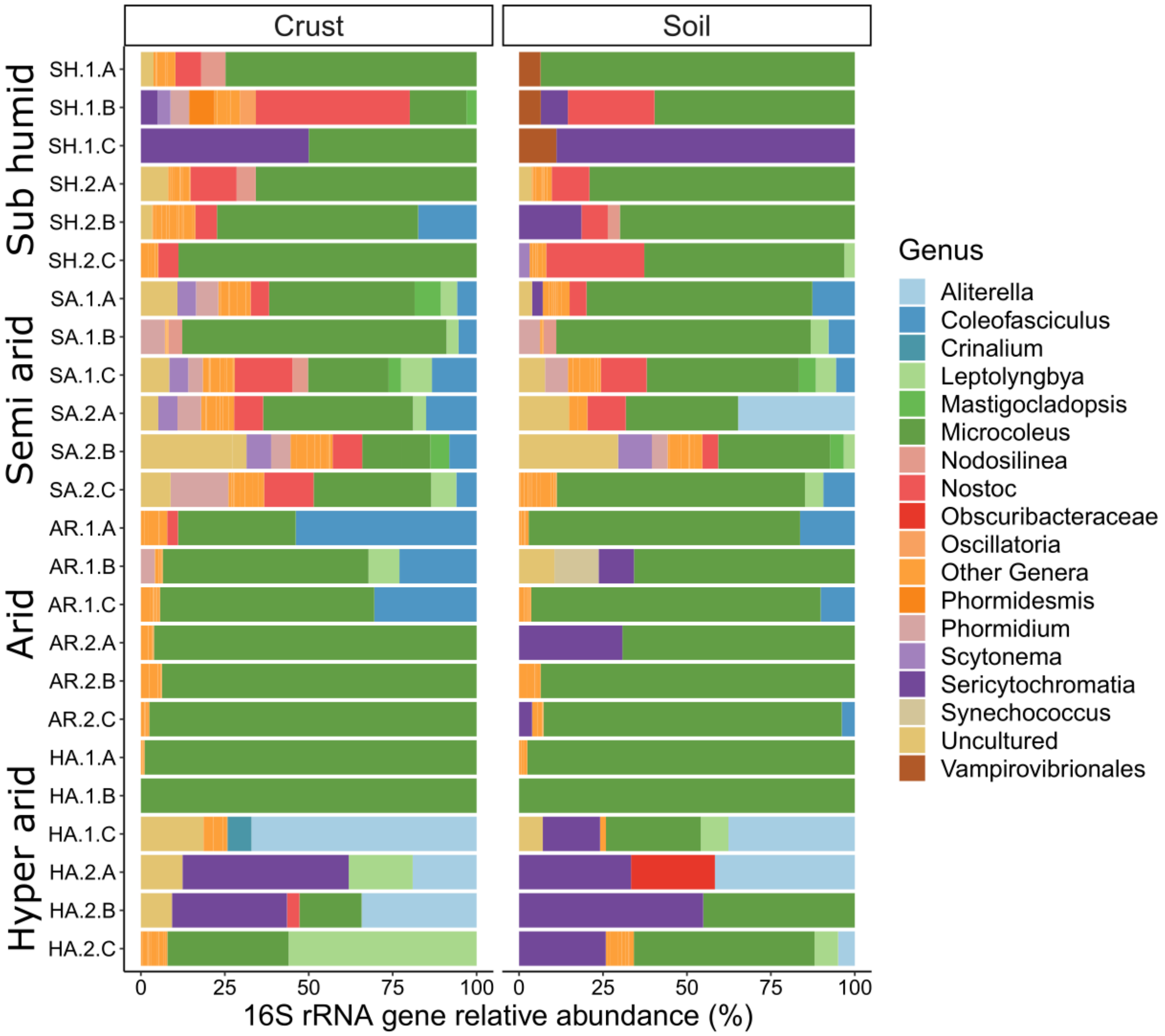

Figure S5. Eukaryotic community composition, estimated richness, and beta diversity based on metagenomic 18S rRNA gene sequences. **(a)** Stacked barchart showing the relative abundance of major eukaryotic divisions detected along the aridity gradient (n = 24). **(b)** Boxplots showing estimated richness by sample type and climatic zone. **(c)** Beta diversity (Bray-Curtis dissimilarity) PCoA ordinations visualising differences by sample type and climatic zone.

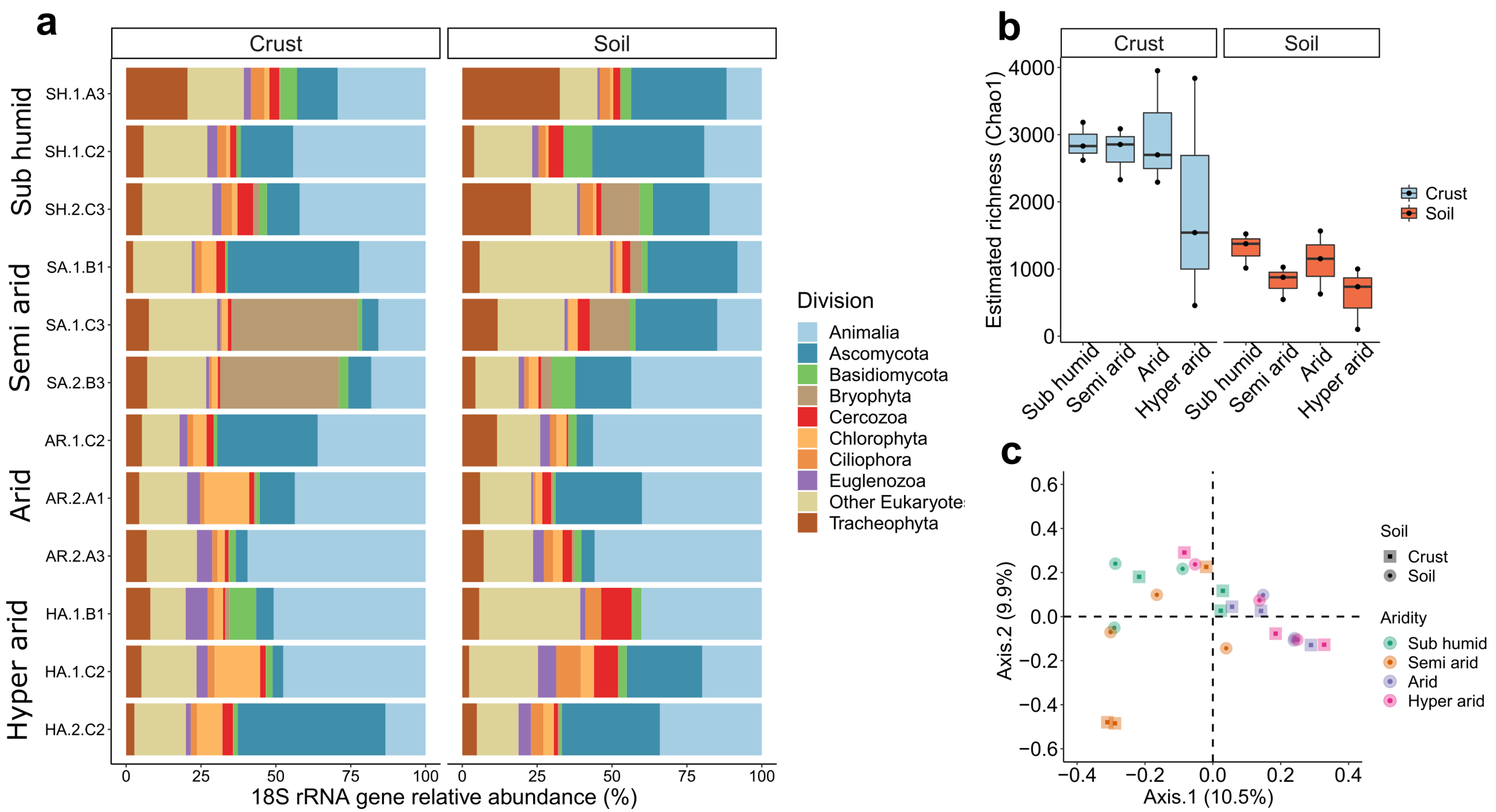

a
Crust
Soil
Sub humid
Semi arid
Arid
Hyper arid
SH.1.A3
SH.1.C2
SH.2.C3
SA.1.B1
SA.1.C3
SA.2.B3
AR.1.C2
AR.2.A1
AR.2.A3
HA.1.B1
HA.1.C2
HA.2.C2
0 25 50 75 100
18S rRNA gene relative abundance (%)
Division
Animalia
Ascomycota
Basidiomycota
Bryophyta
Cercozoa
Chlorophyta
Ciliophora
Euglenozoa
Other Eukaryotes
Tracheophyta
b
Crust
Soil
Estimated richness (Chao1)
0
1000
2000
3000
4000
Sub humid
Semi arid
Arid
Hyper arid
Crust
Soil
c
Axis.1 (10.5%)
Axis.2 (9.9%)
−0.4 −0.2 0.0 0.2 0.4
0.6 0.4 0.2 0.0 −0.2 −0.4 −0.6
Soil
Crust
Soil
Aridity
Sub humid
Semi arid
Arid
Hyper arid

Figure S6. Metabolic capabilities of biocrust and topsoil microbial communities sampled along the aridity gradient. **(a)** Heatmap showing the abundance of genes based on metagenomic short reads. The percentage of the total community predicted to encode at least one of each gene for a process is shown, based on normalization to single-copy marker genes. **(b)** Dot plot showing the metabolic potential of the 68 metagenome-assembled genomes (MAGs). The size of each point represents the number of genomes in each phylum that encode the gene of interest and the shading represents the average genome completeness.

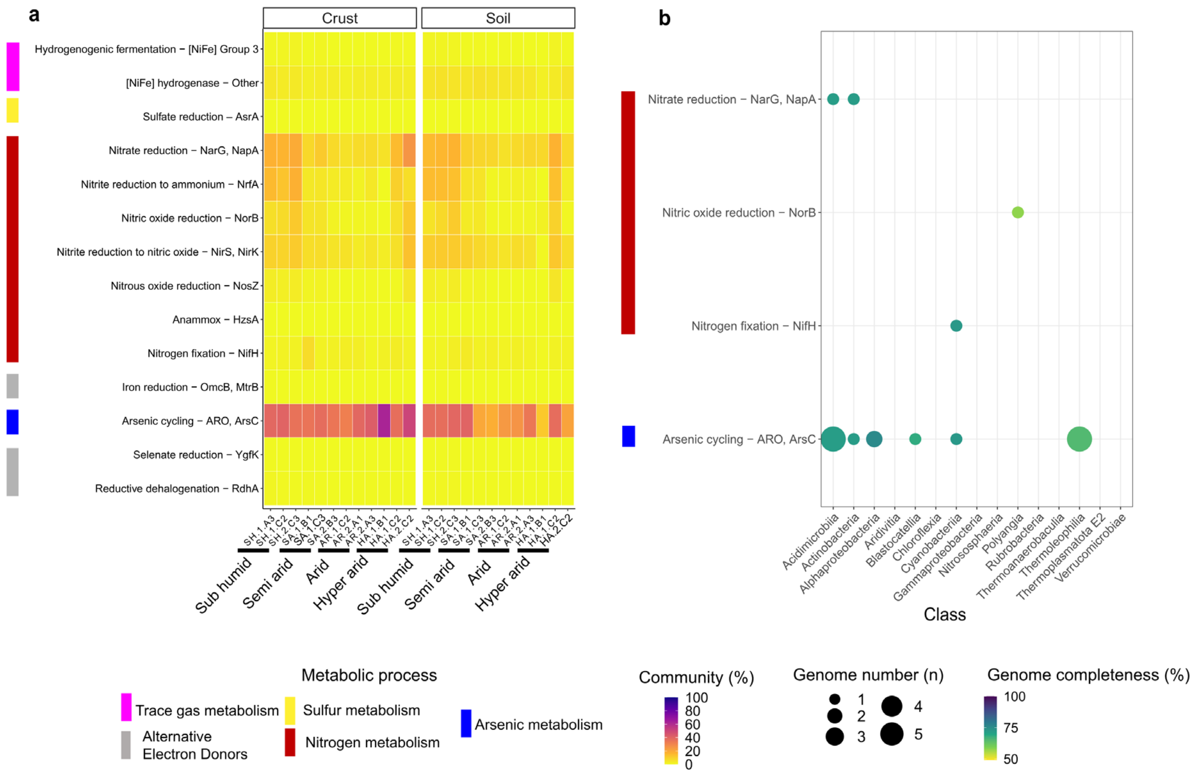

Figure S7. Linear models showing the correlation between physicochemical variables and the community proportion predicted to encode **(a)** group 1h and 1l [NiFe]-hydrogenases, and **(b)** type IA, IB, and IE RuBisCO. Shown are the subset of six uncorrelated edaphic chemical drivers measured along the aridity gradient. R^2 values show the proportion of variance in the H_2 oxidation rates explained by the environmental predictor and shaded ribbons represent 95% confidence intervals.

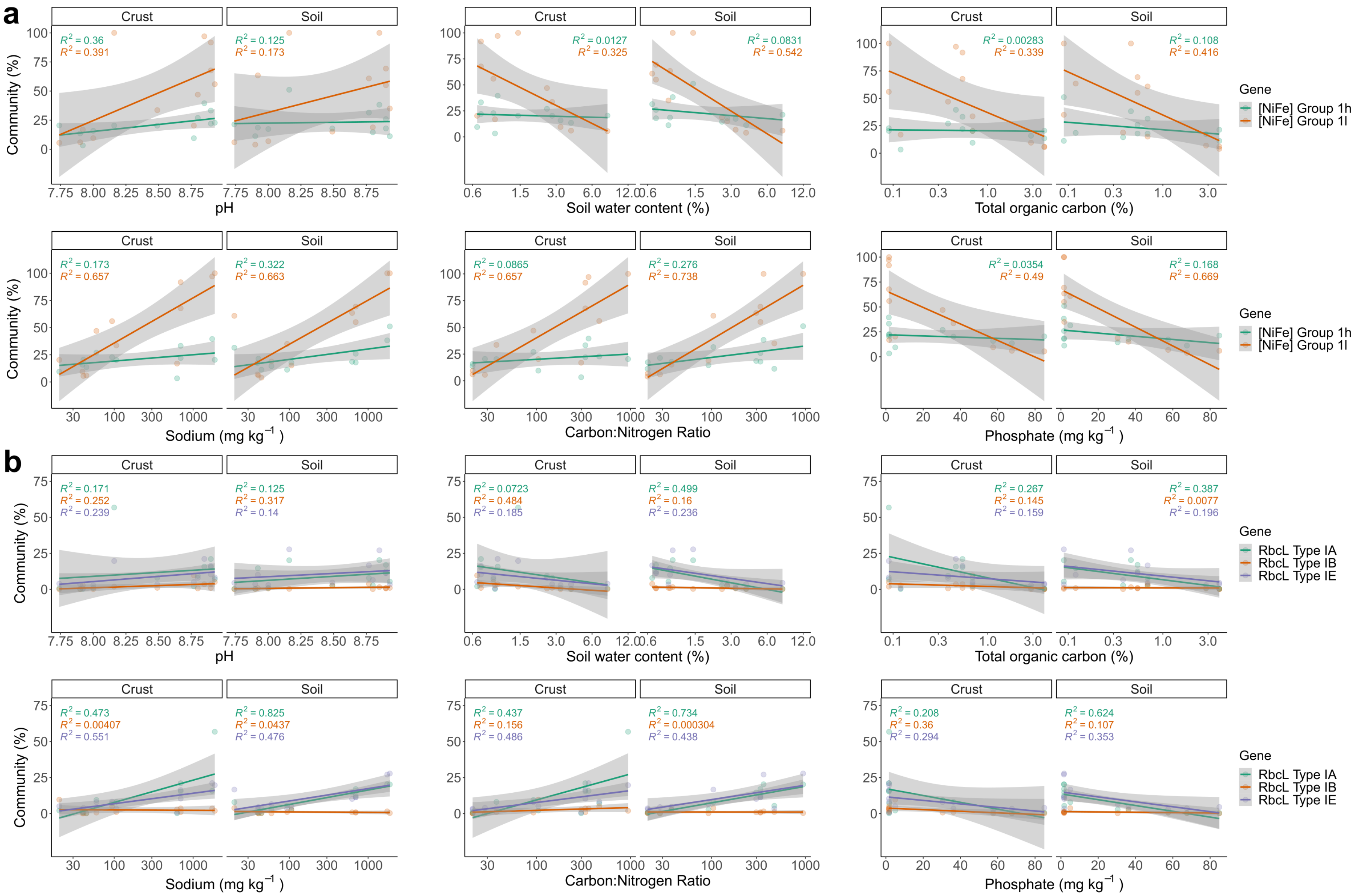

a
Crust
Soil
R^2 = 0.36
R^2 = 0.391
R^2 = 0.125
R^2 = 0.173
Community (%)
pH
R^2 = 0.0127
R^2 = 0.325
R^2 = 0.0831
R^2 = 0.542
Soil water content (%)
R^2 = 0.00283
R^2 = 0.339
R^2 = 0.108
R^2 = 0.416
Total organic carbon (%)
Gene
[NiFe] Group 1h
[NiFe] Group 1l
R^2 = 0.173
R^2 = 0.657
R^2 = 0.322
R^2 = 0.663
Sodium (mg kg^{-1})
R^2 = 0.0865
R^2 = 0.657
R^2 = 0.276
R^2 = 0.738
Carbon:Nitrogen Ratio
R^2 = 0.0354
R^2 = 0.49
R^2 = 0.168
R^2 = 0.669
Phosphate (mg kg^{-1})
b
R^2 = 0.171
R^2 = 0.252
R^2 = 0.239
R^2 = 0.125
R^2 = 0.317
R^2 = 0.14
R^2 = 0.0723
R^2 = 0.484
R^2 = 0.185
R^2 = 0.499
R^2 = 0.16
R^2 = 0.236
R^2 = 0.267
R^2 = 0.145
R^2 = 0.159
R^2 = 0.387
R^2 = 0.0077
R^2 = 0.196
RbcL Type IA
RbcL Type IB
RbcL Type IE
R^2 = 0.473
R^2 = 0.00407
R^2 = 0.551
R^2 = 0.825
R^2 = 0.0437
R^2 = 0.476
R^2 = 0.437
R^2 = 0.156
R^2 = 0.486
R^2 = 0.734
R^2 = 0.000304
R^2 = 0.438
R^2 = 0.208
R^2 = 0.36
R^2 = 0.294
R^2 = 0.624
R^2 = 0.107
R^2 = 0.353

Figure S8. Gas chromatography measurements of biological replicate biocrusts (n = 24) and corresponding topsoil samples (n = 24). H_2 uptake is shown for six biological replicates from each climatic zone for soils **(a-b)** and biocrust samples **(c-d)** during dry and wet microcosm incubations. Error bars represent one standard deviation and the blue dotted line shows average global atmospheric H_2 concentrations (0.53 ppm).

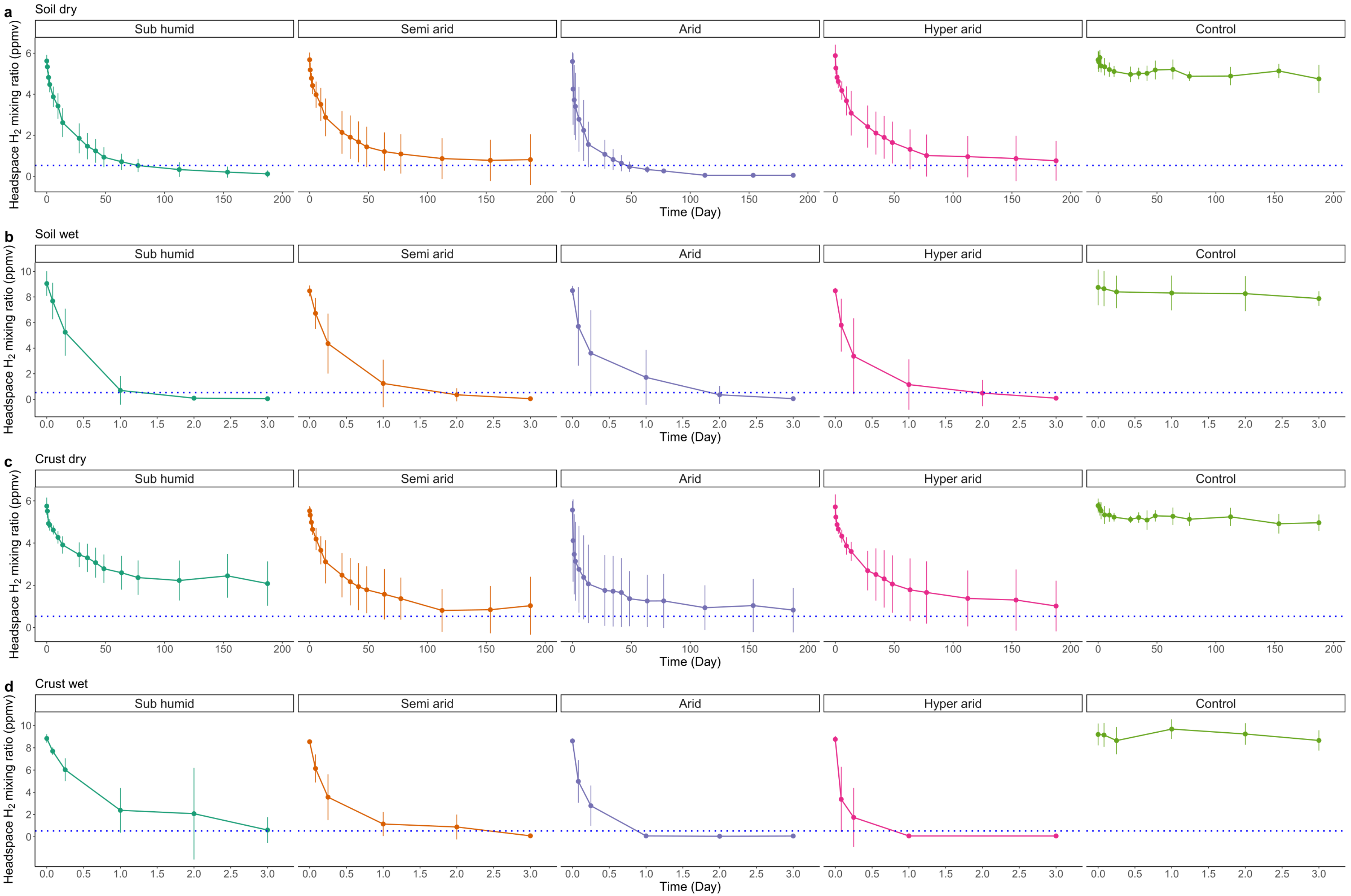
a
Soil dry
b
Soil wet
c
Crust dry
d
Crust wet
Sub humid
Semi arid
Arid
Hyper arid
Control
Headspace H_2 mixing ratio (ppmv)
Time (Day)

Figure S9. Bulk H_2 oxidation and carbon fixation rates in biocrusts and topsoils collected along the aridity gradient. **(a)** Uptake rates of H_2 oxidation measured by gas chromatography of six biological replicates per zone (n = 24) under dry and wet conditions **(b)** Rates of hydrogenotrophic carbon dioxide fixation measured by incorporation of ^{14}C-labelled CO_2 under H_2 enriched and ambient conditions. **(c)** Rates of photosynthetic carbon dioxide fixation measured by incorporation of ^{14}C-labelled CO_2 under light and dark conditions. Carbon dioxide fixation measurements were performed in technical triplicates using a pooled sample from each climatic zone. The box plots show median, upper and lower quartile, and minimum and maximum values.

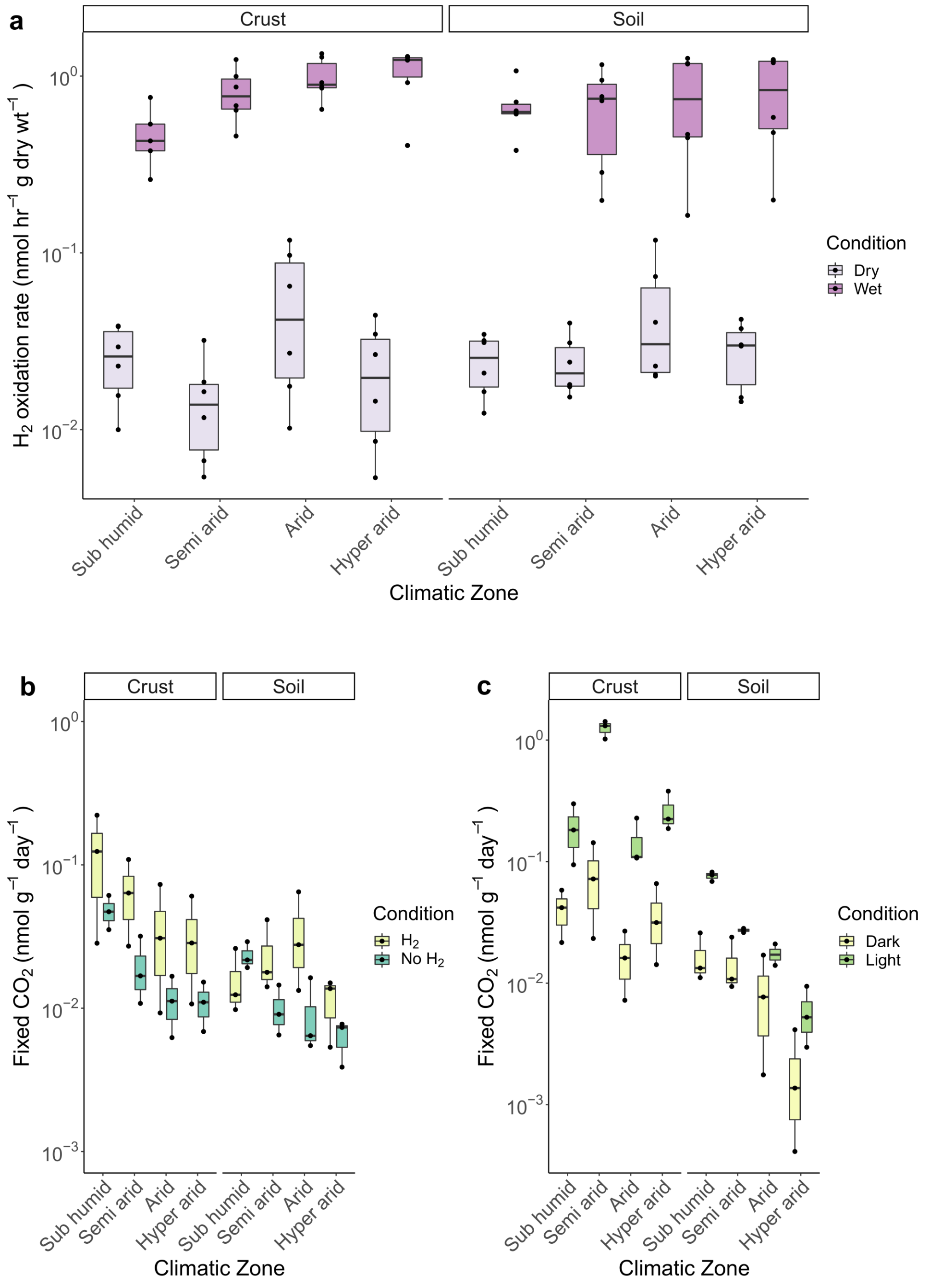

Figure S10. Linear models showing the correlation between H_2 oxidation rates normalised to 16S rRNA gene copy number and the subset of six uncorrelated edaphic chemical drivers measured along the aridity gradient. R^2 values show the proportion of variance in the H_2 oxidation rates explained by the environmental predictor and shaded ribbons represent 95% confidence intervals.

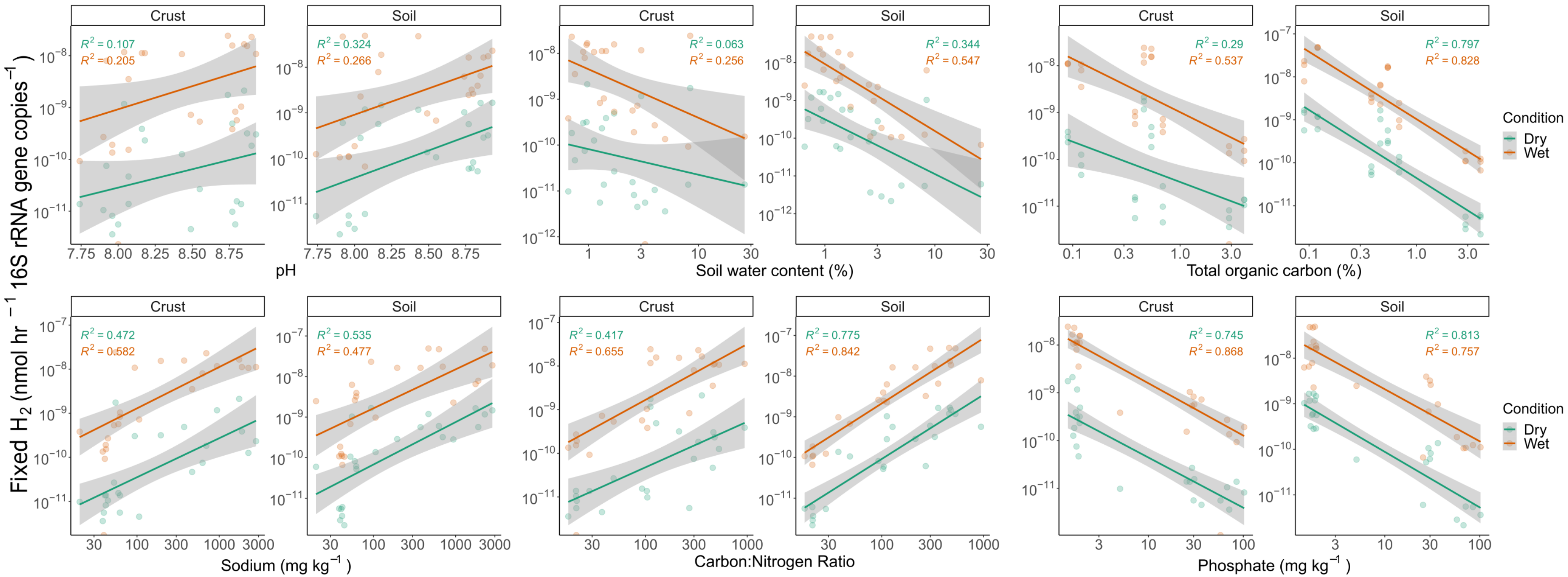

Crust
Soil
Condition
Dry
Wet
$R^2 = 0.107$
$R^2 = 0.205$
$R^2 = 0.324$
$R^2 = 0.266$
$R^2 = 0.063$
$R^2 = 0.256$
$R^2 = 0.344$
$R^2 = 0.547$
$R^2 = 0.29$
$R^2 = 0.537$
$R^2 = 0.797$
$R^2 = 0.828$
$R^2 = 0.472$
$R^2 = 0.582$
$R^2 = 0.535$
$R^2 = 0.477$
$R^2 = 0.417$
$R^2 = 0.655$
$R^2 = 0.775$
$R^2 = 0.842$
$R^2 = 0.745$
$R^2 = 0.868$
$R^2 = 0.813$
$R^2 = 0.757$
pH
Soil water content (%)
Total organic carbon (%)
Sodium (mg kg^{-1})
Carbon:Nitrogen Ratio
Phosphate (mg kg^{-1})
Fixed H_2 (nmol hr^{-1} 16S rRNA gene $copies^{-1}$)

Figure S11. Photosynthetic and hydrogenotrophic carbon fixation activities in biocrusts and topsoils collected along the aridity gradient normalized to 16S rRNA gene copy number. **(a)** Rates of hydrogenotrophic carbon dioxide fixation measured by incorporation of ^{14}C-labelled CO_2 under H_2 enriched and ambient conditions. **(b)** Rates of photosynthetic carbon dioxide fixation measured by incorporation of ^{14}C-labelled CO_2 under light and dark conditions. Carbon dioxide fixation measurements were performed in technical triplicates using a pooled sample from each climatic zone. The box plots show median, upper and lower quartile, and minimum and maximum values.

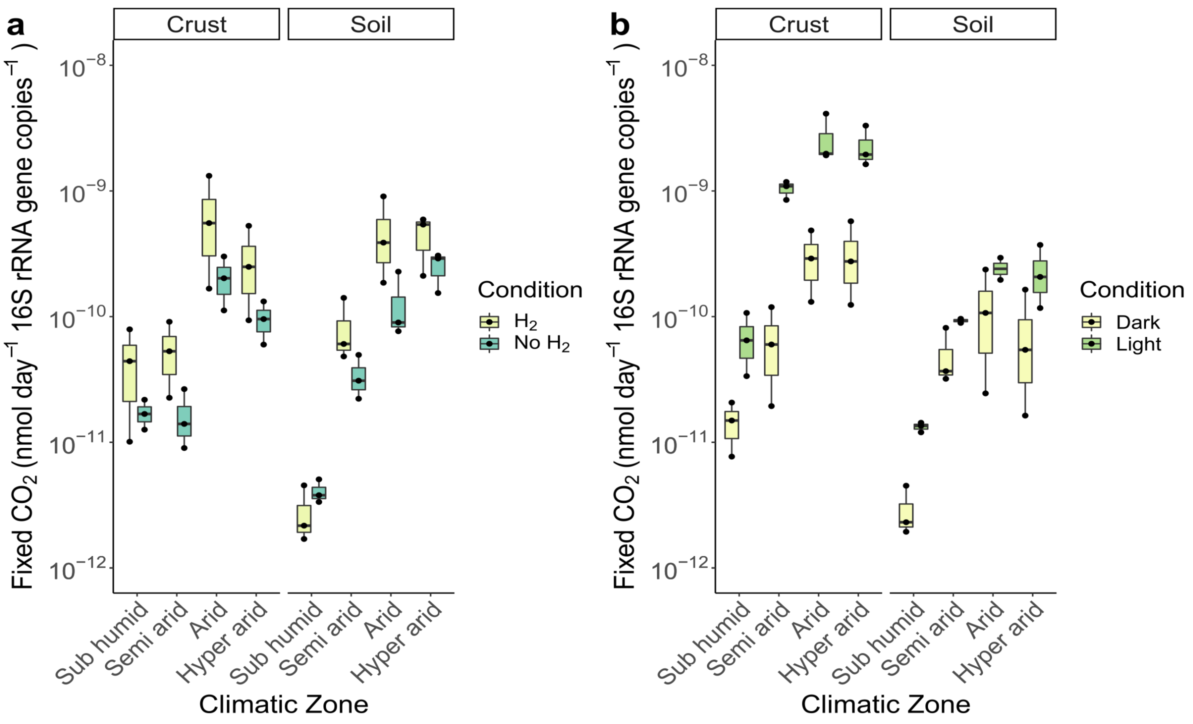

Supplement: Supplementary file 1 — Supplementary material [file 41396_2021_1001_MOESM1_ESM.pdf]
